# Changes in the Biomechanical Properties of Corneal Stromal Lens after Collagen Crosslinking Induced by EDC-NHS

**DOI:** 10.1155/2024/9943458

**Published:** 2024-05-17

**Authors:** Rong Shi, Lijing Wang, Chengpeng Liang, Yu Cheng, Tai Xiang Liu, Xin Luo

**Affiliations:** The Affiliated Hospital of Zunyi Medical University, Zunyi, Guizhou, China

## Abstract

**Introduction:**

To evaluate the changes of lens antidilatation, antiedema, and antienzymolysis ability after different concentrations of 1-ethyl-3-(3-dimethylaminopropyl) carbodiimide and N-hydroxysuccinimide (EDC-NHS)-induced collagen cross-linking.

**Methods:**

Corneal stromal lenticules (*n* = 100) obtained from small incision lenticule extraction (SMILE) procedures were divided into 5 groups: no treatment (control); EDC/NHS (5%/2.5%); EDC/NHS(5%/5%); EDC/NHS (10%/5%); riboflavin and ultraviolet-A light (UVA). Collagen crosslinking was induced using EDC-NHS and UVA. Biomechanical assessments including inflation test, enzymatic degradation resistance, and light transmittance were evaluated posttreatment.

**Results:**

(1) Lenticule apex displacement ranked: control Group > UVA Group > Group (5%/5%) > Group (5%/2.5%) > Group (10%/5%) (Friedman test, *p* < 0.0001). (2) Light transmittance was significantly higher in the crosslinked groups versus control, with EDC/NHS superior to UVA riboflavin. After 15 minutes in PBS, light transmittance decreased due to swelling; however, crosslinked groups maintained significantly higher transmittance versus control. (3) Following crosslinking, enzymatic resistance improved significantly, with the EDC-NHS crosslinking group was significantly better than the UVA cross-linking group.

**Conclusions:**

EDC/NHS crosslinking enhanced lenticule stiffness, antiedema, and enzymatic resistance and without compromising the transparency of the lens. Moreover, EDC/NHS crosslinking efficacy exceeded UVA riboflavin crosslinking in improving lenticule biomechanical properties.

## 1. Introduction

Small incision lenticule extraction (SMILE) is a technique that corrects myopia by extracting a stromal lenticule from the corneal stroma. In recent years, the potential for reuse of extracted lenticules has been extensively investigated [1] for applications including tissue defect repair (corneal ulcer, perforation, dermoid tumors), hyperopia and presbyopia correction, and treatment of corneal ectasia (pellucid marginal degeneration, iatrogenic keratectasia, and keratoconus). However, the thin profile and inherent low stiffness and fragility of individual lenticules have precluded effective utilization of a substantial number of extracted corneal stromal lenticules. We hypothesize that collagen crosslinking may solve the shortcomings of the lens such as low hardness and weak strength.

Collagen crosslinking is the main method to improve the biomechanical properties of the lens. Riboflavin combined with ultraviolet light (UVA) for corneal collagen crosslinking has been widely used in clinical practice [2]. 1-Ethyl-3-(3-dimethylaminopropyl) carbodiimide (EDC) is a water-soluble carbodiimide and a major chemical crosslinking agent. EDC is often combined with N-hydroxysuccinimide (NHS) to augment crosslinking efficiency [3, 4]. The putative mechanism entails EDC-mediated reaction with carboxyl groups on aspartic and glutamic acid residues within and between collagen molecules to form an O-acylisourea intermediate, which can subsequently react with additional collagen carboxyls to form ester bonds. NHS participates as an intermediate reagent, forming amides upon reaction with amines during the crosslinking process. EDC/NHS increases covalent bonds between collagen fibrils, thereby enhancing mechanical strength and enzymatic degradation resistance of collagenous tissues. Our preliminary experiments demonstrated that EDC/NHS-induced vitreal collagen crosslinking can potentiate vitreal tensile strength. We therefore hypothesize EDC/NHS may ameliorate lenticule biomechanical properties by promoting crosslink formation between collagen molecules of the corneal stromal lenticules, which has yet to be reported.

In this study, we will perform collagen crosslinking of corneal stromal lenticules utilizing varying concentrations and ratios of EDC/NHS to delineate the effects of different EDC/NHS levels on lenticule biomechanics and define optimal dose-response relationships. The ultimate goal is to provide novel evidence to effective utilization of the wealth of lenticules derived from SMILE procedures.

## 2. Materials and Methods

### 2.1. Tissue Acquisition and Grouping

Small incision lenticule extraction (SMILE) procedures were performed using the VisuMax 3.0 femtosecond laser system (CarlZeiss Meditec, Jena, Germany). SMILE cap and lenticule parameters were set as follows: lenticule thickness of 120 *μ*m, lenticule diameter of 6.5 mm, and SMILE incision width of 2 mm. After confirming the completion of the SMILE incision and scanning of the lenticule under the surgical microscope, the lenticule was separated from the anterior and posterior corneal surfaces using specialized surgical instruments, and it was extracted. Extracted corneal lenticules were gently transferred from the surgical field and fully immersed in the glycerol solution. Patient gender, age, and preoperative refractive error were recorded for the source of each lenticule. Intact lenticules ranging from −4D to −8D were obtained (*n* = 100).

Lenticules were randomly divided into 5 groups (*n* = 20/Group). Normal control, 5% EDC + 2.5% NHS, 5% EDC + 5% NHS, 10% EDC + 5% NHS, UVA group. The lenticules, with refractive power from −4D to −8D, were sorted accordingly and divided into sections of 5 lenticules each, which were then randomly assigned to the different groups.

### 2.2. Lenticule Crosslinking

EDC/NHS crosslinking: three EDC/NHS-glycerol solutions of varying concentrations were prepared: 5% EDC/2.5% NHS, 5% EDC/5% NHS, and 10% EDC/5% NHS(EDC & NHS: Sigma Aldrich; Glycerol: Solarbio). 150 *μ*l of each EDC/NHS-glycerol solution was added to separate wells of a 96-well plate. Lenticules stored in glycerol were then transferred into the corresponding treatment wells. They were incubated at 4°C for 48 hours to ensure completion of the crosslinking reaction. UVA crosslinking: lenticules were removed from sodium hyaluronate solution, washed in phosphate buffered saline (PBS) to remove the hyaluronate, and then soaked in 0.1% riboflavin solution for 25 mins. In a dark room at 20°C, a 370 nm UV lamp (TIANHUI Electronics Co., LTD, Zhuhai, China) was fixed to a stand and surrounded by ice packs to prevent overheating during irradiation. UV intensity was monitored with a UV340A Ultra-Violet Light Meter (Lutron) to maintain 3 mW/cm^2^ during 30 mins of continuous UV irradiation of the riboflavin-soaked lenticules. One drop of riboflavin solution was added every 3 mins during irradiation. After irradiation, lenticules were stored in glycerol at 4°C for 24 h.

### 2.3. Inflation Testing

**Figure 1:**
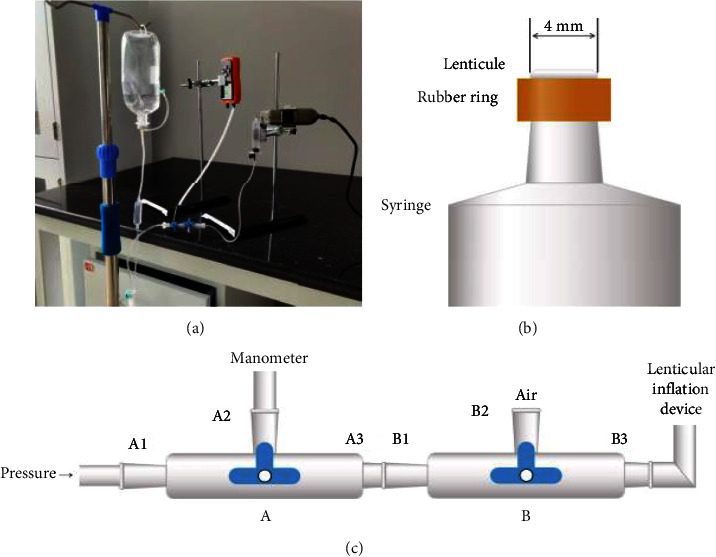
Schematic diagram of the inflation testing setup. (a) Overall inflation system; (b) inflation chamber and lenticule mounting; (c) pressure control valves.

Inflation testing was performed using a custom-built device consisting of four main components: a water reservoir bottle, pressure gauge, inflation chamber, and microscopic camera (Figures 1(a) and 1(b)).

The system was connected and filled with water, removing any air bubbles. Lenticules were washed in PBS to remove glycerol or sodium hyaluronate. The water level of the water reservoir bottle and three-way stopcock were controlled (Figure 1(c)): (1) a certain water level was maintained in B2, B1 was closed, and the lenticule was centered on the syringe nozzle via a rubber ring to ensure a smooth, unwrinkled surface; (2) the syringe and camera were fixed to stands with A3 and B2 closed; (3) with A1 and B3 closed, the three-way stopcock was raised to align B2 water level parallel to the lenticule surface, and the pressure gauge was positioned to read 0 ± 0.2 mmHg; (4) with A3 and B2 closed, the water reservoir bottle was adjusted to 0 ± 0.2 mmHg on the pressure gauge; (5) valve A was opened, B2 was closed, and lenticule apex displacement was measured at incremental pressures of 0, 3, 6 mmHg, up to 51 ± 0.2 mmHg.

### 2.4. Wavelength scanning

Glycerol alone was used as a blank control. An appropriate amount of glycerol was added to a cuvette, and the lenticule was attached to a fixed position on the cuvette to allow light transmission (for the riboflavin crosslinking group, sodium hyaluronate was removed by PBS wash before transferring to glycerol to remove water prior to measurement). Light transmittance was measured using a UV spectrophotometer (UH5300, Hitachi, Japan) with wavelength scans from 380 to 780 nm at a rate of 1200 nm/min and intervals of 2.5 nm.

### 2.5. Swelling experiments

Residual glycerol or sodium hyaluronate was removed from lenticule surfaces by PBS wash. Lenticules were then soaked in PBS for 15 mins prior to measuring light transmittance.

### 2.6. Enzymatic Degradation Experiments

10 mg Collaenase I, Invitrogen17100-017, Biosharp, dissolved in 10 ml PBS solution. 150 *μ*l of collagenase solution was added to each well of a 96-well plate. Lenticules were washed in PBS to remove surface glycerol, then transferred separately into the collagenase solution wells, and incubated at 25°C in a water bath. Mass was measured hourly, with lenticules removed, flattened on the absorbent paper to gently blot surface water, and then weighed. After 5 measurements, fresh collagenase solution was replaced. Lenticules were considered amorphous solids when they are no longer able to be grasped intact from the paper.

### 2.7. Statistical Analysis

Statistical analysis and figure generation were performed using R version 4.2.1.

## 3. Results

### 3.1. EDC/NHS Collagen Crosslinking Improves Lenticule Resistance to Inflation

**Figure 2:**
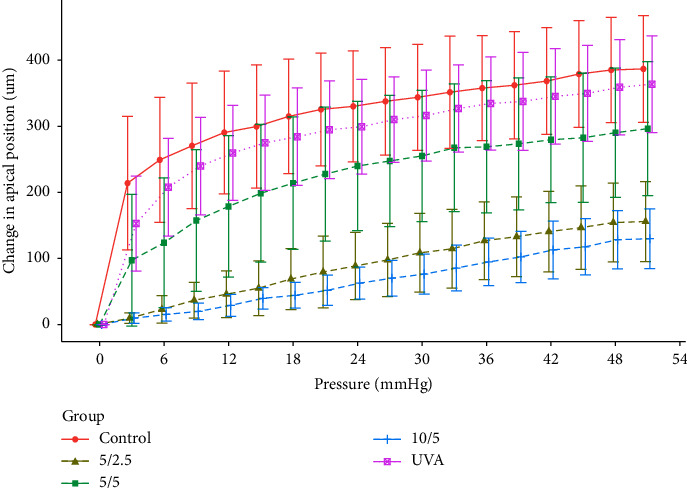
Comparison of displacement changes of lens vertex in each group with increasing pressure.

With increasing pressure, lenticule inflation occurred and apex displacement increased accordingly. At 51 mmHg pressure, mean apex displacement ranked from largest to smallest: control (386.8 ± 80.8 *μ*m), UVA (363.6 ± 73.2 *μ*m), 5/5 (296.4 ± 101.4 *μ*m), 5/2.5 (155.8 ± 60.6 *μ*m), 10/5 (129.8 ± 45.2 *μ*m), with significant differences among groups (Friedman test, *p* < 0.0001). Specifically, the UVA group differed significantly from the 5/2.5 and 10/5 groups (Conover's test, *p* < 0.05), and the control group differed significantly from the 5/2.5 and 10/5 groups (Conover's test, *p* < 0.01).Compared to control and riboflavin crosslinking groups, EDC/NHS crosslinking significantly enhanced resistance to lenticule inflation. Inflation resistance ranked 10/5 > 5/2.5 >> 5/5 (Figure 2).

### 3.2. EDC/NHS Collagen Crosslinking Does Not Compromise Lenticule Light Transmittance

**Figure 3:**
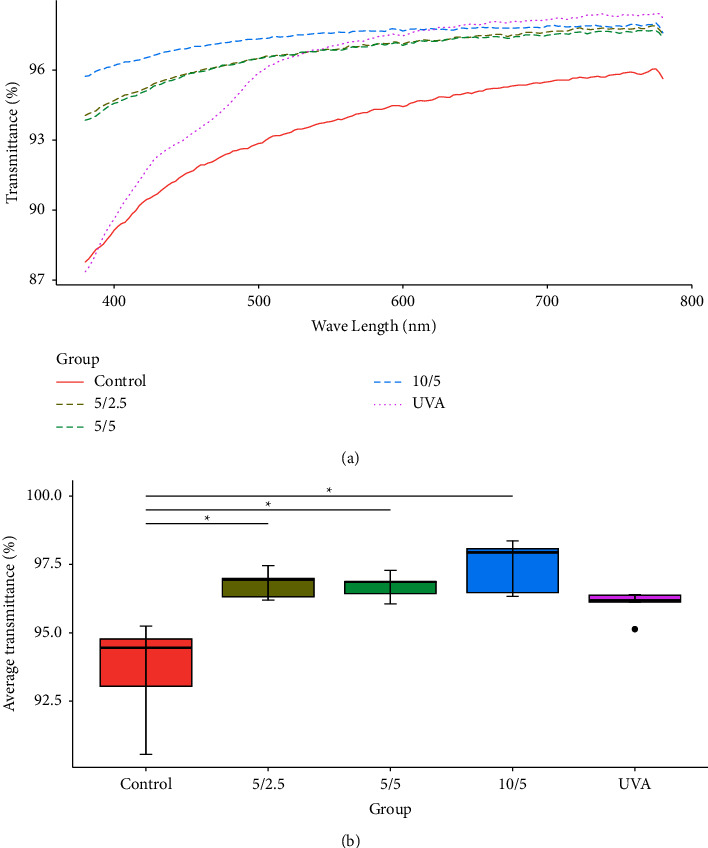
(a) Lenticule light transmittance comparison in the visible spectrum; (b) comparison of average transmittance. ^*∗*^indicates significant difference *p* < 0.05 (BWS all-pairs test).

Lenticule light transmittance in the visible spectrum (380−780 nm) is shown in Figure 3(a). The crosslinked groups had significantly higher transmittance compared to control. The riboflavin crosslinking group showed a more pronounced decrease in transmittance from 380 to 500 nm (Figure 3(a)).

The average transmittance was the mean transmittance value across all detected wavelengths within the detection range for each lenticule. The differences in average transmittance among groups were statistically significant (Kruskal–Wallis test, *p*=0.003). The control group differed significantly from all three EDC/NHS crosslinking groups, while differences between the treatment groups were not statistically significant. EDC/NHS crosslinking did not compromise and rather enhanced the light transmittance of the lenticules (Figure 3(b)).

### 3.3. EDC/NHS Collagen Crosslinking Can Improve the Anti-Edema Ability of Lens

Since the lenticules were soaked in glycerol, it was difficult to perform conventional swelling experiments by comparing mass differences before and after water immersion. Corneal swelling leads to decreased light transmittance; thus, changes in transmittance can indirectly indicate lenticule water absorption, with lower transmittance suggesting greater swelling capacity.

**Figure 4:**
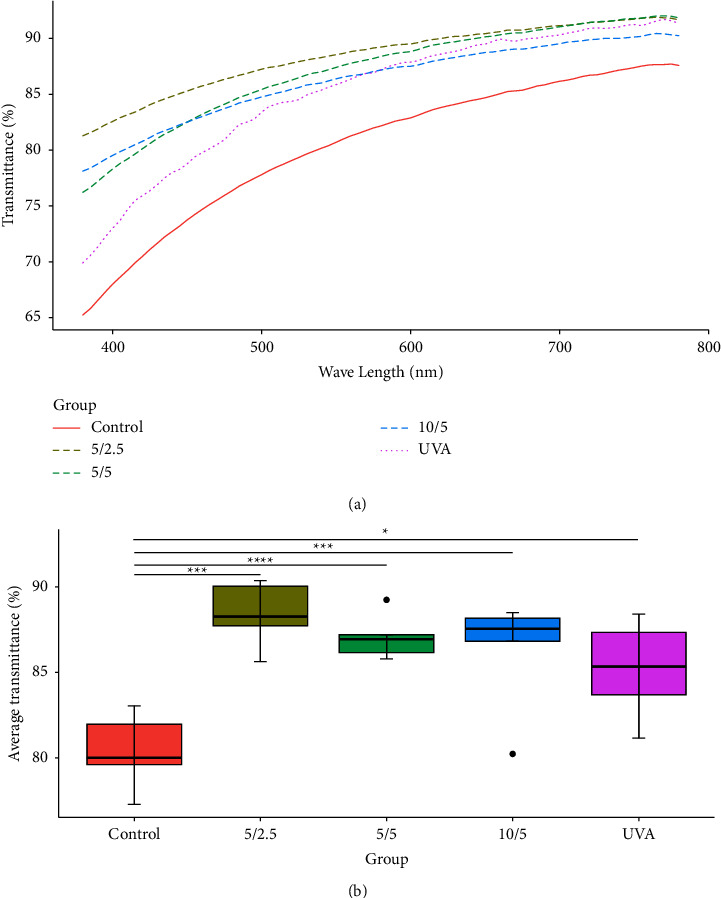
(a) Comparison of lenticule transmittance after soaking in PBS for 15 mins; (b) comparison of average transmittance after soaking in PBS for 15 mins. ^*∗*^*p* < 0.05, ^*∗∗∗*^*p* < 0.001, ^*∗∗∗∗*^*p* < 0.0001 by *T*-test.

After soaking in PBS for 15 mins, lenticule swelling caused decreased light transmittance. The crosslinked groups had significantly higher transmittance across the visible spectrum compared to the control (Figure 4(a)).

The differences in average transmittance among groups were statistically significant (one-way ANOVA, *p* < 0.0001). Specifically, the control group differed significantly from all crosslinking groups, while differences between the other groups were not statistically significant (Figure 4(b)).

Compared to preimmersion, the crosslinking groups showed a smaller decrease in transmittance after soaking in PBS versus control. However, the differences in change between the groups post-PBS immersion were not statistically significant (one-way ANOVA, *p*=0.064). After crosslinking, lenticule transmittance post-PBS immersion was higher than the control, indicating less swelling versus control (Table 1).

### 3.4. EDC/NHS Collagen Crosslinking Enhances Lenticule Enzymatic Degradation Resistance

The lenticules gradually degraded upon immersion in the collagenase solution. Lenticules with different treatments showed differing degradation patterns and rates. The control group degraded most rapidly, completely losing basic shape by 4 h. The riboflavin crosslinking group showed a steady mass decrease, then abruptly lost basic shape at 5 h. The three EDC/NHS crosslinking groups exhibited a steady mass decline over time, with subtle differences in reduction rate and the order of degradation rate being 5/2.5 ≈ 5/5 > 10/5.

**Figure 5:**
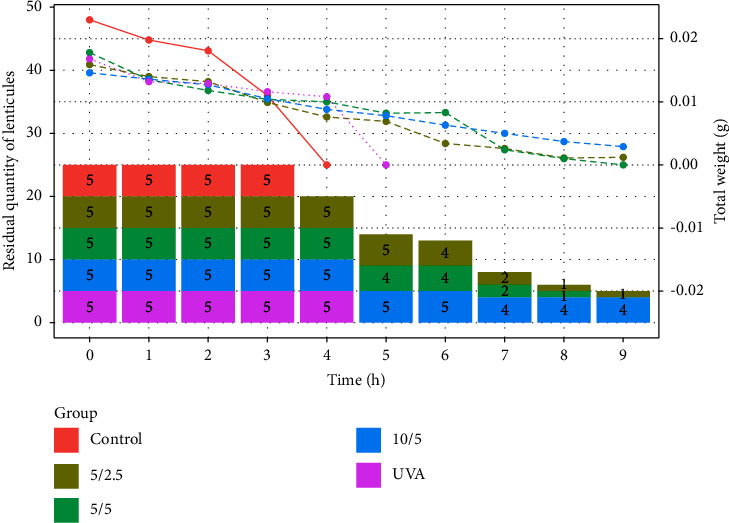
Soaking time in collagenase solution on the *x*-axis, with the *y*-axis showing the number of lenticules in each group maintaining basic shape, and total mass of 5 lenticules per group.

Slower degradation indicates greater enzymatic resistance. Both riboflavin and EDC/NHS crosslinking improved lenticule enzymatic resistance. Under the experimental concentrations, EDC/NHS crosslinking was more effective than riboflavin crosslinking, with the 10/5 group demonstrating the strongest enzymatic resistance among the three EDC/NHS concentrations (Figure 5).

## 4. Discussion

### 4.1. EDC/NHS Crosslinking Enhanced Lenticule Stiffness and Resistance to Inflation to a Much Greater Extent than Riboflavin/UVA Crosslinking

This difference likely stems from the ability of EDC/NHS to catalyze formation of covalent bonds between collagen molecules versus the proposed noncovalent mechanisms of riboflavin/UVA.

In this study, following EDC/NHS crosslinking, pronounced increases in lenticule stiffness were observed, with the 5/2.5 and 5/5 groups displaying significantly reduced apex displacement in inflation testing compared to control and riboflavin/UVA groups. EDC/NHS crosslinking enhanced lenticule resistance to inflation to a far greater extent than riboflavin/UVA crosslinking, potentially attributable to differences in the molecular mechanisms of the two crosslinking modalities. While widely utilized clinically, the molecular events underlying riboflavin/UVA crosslinking remain incompletely defined. The primary hypothesis postulates riboflavin facilitates generation of singlet oxygen and other reactive oxygen species upon UVA irradiation to promote formation of covalent bonds between collagen fibrils. However, using surface enhanced Raman spectroscopy, Melcher et al. [5] detected minimal differences in covalent bonds before and after UVA exposure, suggesting that noncovalent effects may be integral to riboflavin/UVA crosslinking efficacy. In contrast, EDC/NHS crosslinking catalyzes formation of covalent amide bonds between carboxyl and amine groups. Thus, disparities in strength between noncovalent interactions and covalent bonds may underlie the differences in crosslinking efficacy observed between these two modalities.

### 4.2. The 5%/2.5% and 10%/5% EDC/NHS Groups Showed Superior Inflation Resistance Compared to 5%/5%

This is potentially due to the ability of higher NHS concentrations to promote intramolecular crosslinking within collagen triple helices, impacting mechanical properties.

In the present study, the EDC/NHS ratio substantially influenced crosslinking efficacy, with the 5/2.5 and 10/5 groups demonstrating comparable lenticule inflation resistance exceeding that of the 5/5 group. Theoretically, higher relative NHS content should better “protect” O-acylisourea intermediates from hydrolysis to enable increased covalent bond formation and consequently greater material stiffness after crosslinking. However, both our study and others have found paradoxically reduced material hardness with excessively high NHS levels [6, 7]. In the enzymatic degradation experiments, slower degradation likely reflects a higher degree of collagen crosslinking. Our observation of similar degradation rates between the 5/5 and 5/2.5 groups suggests potentially comparable extents of collagen crosslinking in these groups. Thus, the inferior resistance to inflation of the 5/5 group could arise from divergent crosslinking locations rather than densities.

Gratzer and Lee [4] reported that collagen gels crosslinked without controlled pH exhibited lower bend resistance compared to gels crosslinked precisely at pH 5.5. In the uncontrolled pH condition, the pH rapidly increased from 5.7 to 5.8 and then gradually decreased to 5.4. However, the enzymatic resistance was similar between the two groups. They proposed this resulted from altered crosslinking loci with different pH conditions. Crosslinking at pH 5.5 increased thermal stability yet yielded more unmodified lysine residues, indicating greater intramolecular crosslinks between collagen triple helices, while uncontrolled pH favored intermolecular crosslinks between collagen molecules, collagen and glycoproteins, and between collagen lamellae. Distinctions in thermal stability primarily manifested early during crosslinking, overlapping with the higher pH (>5.5) stage of the uncontrolled pH group, suggesting that higher pH promotes intermolecular collagen crosslinking. Analogously, in our study, the 5/5 group showed weaker inflation resistance compared to the 5/2.5 group, potentially attributable to more collagen intramolecular crosslinking, conferring reduced bend resistance. The hypothesized mechanism is that the weak acid NHS (pKa 6.0 [8]) lowered and buffered pH at higher relative content, thereby promoting collagen intramolecular crosslinking.

### 4.3. EDC/NHS Crosslinking Did Not Compromise and Even Enhanced Lenticule Light Transmittance Compared to Controls

This improvement may result from increased collagen fibrillar alignment after crosslinking.

Relative to noncrosslinked controls, light transmittance was not compromised and instead improved for all crosslinked groups, potentially attributable to enhanced collagen fiber alignment regularity postcrosslinking [9], which impacts corneal transparency [10]. The marked transmittance decline of the riboflavin crosslinking group in the near UV spectrum likely stems from residual riboflavin absorption peaks at 371 nm and 442 nm [11]. However, at 7 weeks following riboflavin crosslinking in rabbit corneas, near UV transmittance remained approximately 20% lower [12], the mechanisms underlying which warrant further investigation.

### 4.4. Crosslinked Groups Exhibited Higher Transmittance after PBS Immersion, Indicating Reduced Swelling Capacity

This resistance may result from interfiber crosslinking occurrences.

Corneal hydration or swelling reduces optical transparency, with greater hydration associated with lower transmittance [13]. Thus, alterations in lenticule light transmittance can reflect the extent of hydration. Given the significant preswelling transmittance differences between noncrosslinked and crosslinked groups, the pre- to postswelling transmittance change was compared. Although no significant differences were detected, the crosslinked groups universally demonstrated smaller transmittance changes versus the noncrosslinked controls, suggesting that crosslinking may mitigate lenticule swelling capacity. Furthermore, corneal hydration exhibits a robust linear relationship with the square of collagen interfibrillar spacing [14]. The 5/2.5 group with the lowest hydration accordingly exhibited the smallest collagen interfibrillar spacing, circumstantially evidencing greater interfiber crosslinking occurrences.

### 4.5. EDC/NHS Crosslinking, Especially 10%/5%, Greatly Improved Resistance to Collagenase Degradation Compared to Noncrosslinked Controls

The covalent crosslinks formed by EDC/NHS are proposed to better maintain collagen fibrillar connectivity despite enzymatic cleavage.

The lenticules exhibited gradual degradation under protease activity, with distinct patterns between the groups. In noncrosslinked and riboflavin crosslinked groups, basic shape was progressively lost over time, with substantial residual matrix at later timepoints that could not be grasped. By contrast, EDC/NHS crosslinked groups largely retained their circular geometry while mass steadily declined. Theoretically, collagenase cleavage produces fragments of varying lengths, with smaller detached peptides dissolving to cause the gradual mass reduction. Larger segments experience cohesive forces to remain intact until additional degradation occurs. As collagen fibers incrementally fracture, the lenticule increasingly cannot maintain its basic shape. At a certain degree, the residual collagen can no longer withstand forceps grasping. EDC/NHS crosslinked groups preserved basic shape longer, likely because crosslinks formed covalent bonds to retain fiber connectivity despite enzymatic cleavage at susceptible loci.

Watanabe-Nakayama et al. [15] extensively delineated the collagenase I degradation mechanism, encompassing binding, cleavage, and fragment drop-off stages. Intermolecular collagen interactions impede collagenase engagement. Thus, crosslinking augments intermolecular forces to confer enhanced enzymatic resistance compared to noncrosslinked controls. More robust crosslink-mediated intermolecular interaction strength imparts greater resistance. The markedly enhanced enzymatic resistance of the 10/5 group accordingly suggests this group formed the most extensive covalent bonds.

## 5. Limitations

### 5.1. In Vitro Study

This is an in vitro study using extracted lenticules. The effects of EDC/NHS crosslinking in vivo or its biocompatibility with ocular tissues are not evaluated.

### 5.2. Sample Size

The number of lenticules per experimental group (*n* = 20) is relatively small, which could impact the statistical power of the analyses.

### 5.3. Single Tissue Source

All lenticules were obtained from a single clinical center. Broader sampling from multiple centers could improve the generalizability of the findings.

### 5.4. Limited Long-Term Evaluation

The study does not assess the long-term stability or potential degradation of the crosslinked lenticules over extended time periods.

### 5.5. Mechanism Not Fully Elucidated

While the study proposes possible mechanisms, the precise molecular events underlying the differential effects of varied EDC/NHS concentrations are not conclusively determined.

## 6. Conclusions

This study elucidated the effects of varied EDC/NHS crosslinking protocols on lenticule properties. EDC/NHS crosslinking enhanced lenticule stiffness and enzymatic resistance without compromising optical performance. Crosslinking efficacy depended on the EDC/NHS ratios, enabling customization of desired biomechanical properties by modulating relative concentrations.

## Figures and Tables

**Table 1:** Changes in lenticule transmittance before and after PBS immersion.

Group	Before swelling	Before swelling	Variance
Control	93.61 ± 1.90	80.38 ± 2.23	13.23 ± 3.78
5/2.5	96.78 ± 0.52	88.40 ± 1.92	8.38 ± 2.11
5/5	96.70 ± 0.47	87.07 ± 1.34	9.63 ± 1.56
10/5	97.28 ± 1.03	87.76 ± 0.75	9.52 ± 0.52
UVA	96.05 ± 0.52	85.19 ± 2.89	10.86 ± 2.97

## Data Availability

The datasets generated during the current study are available from the corresponding author on reasonable request.

## Supplementary Materials

Supplementary Materials In this study, the preservation methods of corneal stromal lens, collagen crosslinking methods, and inflation testing were based on the previous research results of our team [16] and the research results of Matthew et al. [17].
