# Supplementary material for: Changes in the Biomechanical Properties of Corneal Stromal Lens after Collagen Crosslinking Induced by EDC-NHS
Source: J Ophthalmol. 2024 May 17;2024:9943458. doi: 10.1155/2024/9943458 (PMC11126343; doi:10.1155/2024/9943458)
Supplement: Supplementary Materials — In this study, the preservation methods of corneal stromal lens, collagen crosslinking methods, and inflation testing were based on the previous research results of our team [16] and the research results of Matthew et al. [17]. [file 9943458.f1.zip › Complaince status of smoke free public places law in Nepal manuscript (1).docx]

**Compliance Status of Smoke-Free Public Places Law in Nepal**

**Aditya Shakya^1^, Bhakta Bahadur KC^2^, Rasmita Shrestha^3^**

*^1^ Health Office Ilam, Ministry of Health, Koshi Province, Nepal*

*^2^ National Health Education Information and Communication Center, Kathmandu, Nepal*

*^3^ B.P. Koirala Institute of Health Science, Dharan, Nepal*

**Correspondence:** Aditya Shakya; shakyaditya@gmail.com

**Compliance Status of Smoke-Free Public Places Law in Nepal**

**Aditya Shakya^1^, Bhakta Bahadur KC^2^, Rasmita Shrestha^3^**

*^1^ Health Office Ilam, Ministry of Health, Koshi Province, Nepal*

*^2^ National Health Education Information and Communication Center, Kathmandu, Nepal*

*^3^ B.P. Koirala Institute of Health Science, Dharan, Nepal*

**Correspondence:** Aditya Shakya; shakyaditya@gmail.com

**ABSTRACT**

**Background**: The tobacco epidemic is one of the biggest public health threats the world has ever faced, killing around 8 million people a year. The tobacco control program has been one of the priority programs in Nepal but still satisfactory results haven’t been achieved yet. The main objective of this study was to assess the tobacco smoke-free law compliance in public places of the provincial headquarters of Nepal.

**Methods**: A cross-sectional observational study was conducted in 2019 in 560 public places of seven provincial headquarters of Nepal. Purposive samples were collected from at least 3 different clusters (wards) of each city. Indoor and outdoor locations of public places were observed. Observation tool was adopted from the Guide for conducting compliance studies (John Hopkins School of Public Health). Data were entered in Epi Data v3.1 and analysis was done using IBM SPSS 26.

**Results:** This study showed less use of no-tobacco signage which was 32% in the indoor areas and 13% in the outdoor areas in public places but they were placed in visible areas. Tobacco use in public places is still prevalent and it was higher (40%) in outdoor of public places. Three-fourth (76%) of public places was observed for buying and selling tobacco products within their 100 meter premises. Tourist hotels, public toilets, and bus stops/ticket counters had low tobacco law compliance both in indoor and outdoor areas. Though public vehicles and cinema halls had high tobacco law compliance in indoor areas, law compliance in outdoor areas was low. Province-wise comparison showed there was a similar pattern of tobacco law compliance in the indoor areas but outdoor law compliance was low in Madesh, Bagmati and, Karnali province.

**Conclusion:** It is evident that while progress has been made in certain aspect of tobacco law compliance, there remains a compelling need for comprehensive and targeted interventions to ensure adherence to laws in public places across the country.

**BACKGROUND**

Tobacco is the leading preventable cause of death globally, causing around 8 million deaths annually[[1](#_ENREF_1)]. It is the only legal consumer product that harms both users and those exposed to second-hand smoke. The tobacco epidemic poses a significant public health threat, with more than 7 million deaths directly attributed to tobacco use and approximately 1.3 million deaths resulting from exposure to second-hand smoke [[2](#_ENREF_2)]. Despite its deadly consequences, widespread use persists due to factors such as low prices, aggressive marketing, lack of awareness, and inconsistent public policies[[1](#_ENREF_1)].

Over 80% of the one billion global smokers live in low and middle-income countries, facing the heaviest burden of tobacco-related illness and death[[3](#_ENREF_3)]. By 2030, more than 80% of such deaths are expected in these countries [[1](#_ENREF_1)]. In Nepal, tobacco-related morbidity and mortality is increasing with a 17.1% prevalence of current smokers among adults aged 15-69 [[4](#_ENREF_4), [5](#_ENREF_5)]. Nepal Global Youth Tobacco Survey (GYTS) 2011 showed a doubling in the prevalence of tobacco use among school-going adolescents from 9.4% in 2007 to 20.4% in 2011 [[6](#_ENREF_6), [7](#_ENREF_7)].

The WHO Framework Convention on Tobacco Control (FCTC) was the first step in the global fight against the tobacco epidemic and this multilateral treaty presents a blueprint for countries to reduce both the supply and demand for tobacco [[1](#_ENREF_1)]. Based on the WHO FCTC, Nepal has brought Tobacco Product Control and Regulatory Act, 2011 to make legal provisions to reduce, control, and regulate the import, production, sales, distribution, and consumption of tobacco products but still satisfactory results haven’t been achieved yet [[5](#_ENREF_5), [8](#_ENREF_8)].

The World Health Organization (WHO) acknowledges the importance of prioritizing the protection of individuals from secondhand smoke exposure as a global public health imperative [[2](#_ENREF_2)]. In alignment with this, Nepal has implemented bans on tobacco use in various types of public places[[9](#_ENREF_9)]. The information about tobacco law compliance will be the key for controlling the tobacco epidemic in Nepal which also complements the FCTC 2030 strategy and SDG goal of healthy cities[[10](#_ENREF_10)]. Most of the studies in Nepal have been focused on the prevalence of tobacco use; few have explored its associated factors and none on tobacco law compliance [[11-14](#_ENREF_11)]. The main objective of this study was to assess the tobacco smoke-free law compliance in public places of the provincial headquarters of Nepal.

# **METHODS**

***Study design and setting****:* A cross-sectional observational study was conducted in 16 public places listed in Nepal's Tobacco Control and Regulation Act 2011 in seven provincial headquarters (Biratnagar, Janakpur, Hetauda, Pokhara, Butwal, Surkhet, Dhangadi) of Nepal from May to July of 2019.

***Study size and sampling****:* Considering a 50 % prevalence of tobacco law compliance, 5 % level of significance, 5 % margin of error, and design effect of 1.5, a sample size of 576 was calculated. A total of 560 public places were observed and 2.8% was non-response rate. Public places were observed from at least 3 different wards of each city and a convenient sampling technique was used for the selection of public places.

***Variables****:* Tobacco law compliance was categorized into three categories i.e. low (<50%), medium (50% to 80%), and high (>80%). For airports and tourist hotels that were authorized to have designated areas as smoking zones, 14 indicators for indoor areas and 12 indicators for outdoor area observation were used in the checklist. For the other 14 public places, 9 indicators were used for observation of both indoor and outdoor areas. Tobacco law compliance was examined according to the public place and province.

***Data collection****:* We utilized an observation checklist for tobacco law compliance for conducting compliance studies developed by the Johns Hopkins School of Public Health. The observation checklist was translated into Nepali language and re-translated into the English language. Pretesting was done in public places in Kathmandu District for tool validation. Enumerators were oriented on data collection tools and processes. The observation was done for indoor and outdoor of public places. Formal sectors were observed during official time only and most of the public places were observed during the day time. Enumerators were daily monitored and supervised through phone calls and log sheets.

***Data analysis****:* Data was entered in Epi Data v3.1 and analysis was done using IBM SPSS 26. Descriptive analysis was done by calculating frequency and percentages for categorical variables.

***Ethical consideration****:* Ethical clearance was obtained from the Ethical Review Board (ERB) of the Nepal Health Research Council (NHRC). Written informed consent was obtained from study institutions before data collection. Institutions were given full right not to take part or continue in the study at any time during the study. Confidentiality of the institution's information was maintained.

# **RESULTS**

**Type of public places**

Eighty public places were selected from each province. Table 1 shows that of all the public places selected in the study, public vehicles had the highest percentage with 13.9% and airports had the lowest percentage i.e. 0.7%. Hostel, cinema hall, public toilet, and child care/orphanage/old age house/park have less than average proportion of public places and all other 10 public places have average proportion.

Table 1: Types of public places

| **Public places** | **Number** | **Percentage** |
| --- | --- | --- |
| Hotel/ restaurant | 38 | 6.8 |
| Educational institution/ library | 38 | 6.8 |
| Religious places/ pilgrimage | 38 | 6.8 |
| Government office | 30 | 5.4 |
| Public vehicle | 78 | 13.9 |
| Industry | 42 | 7.5 |
| Health institution | 35 | 6.3 |
| Bus stop/ ticket counter | 41 | 7.3 |
| Hostel | 28 | 5.0 |
| Cinema hall | 23 | 4.1 |
| Child care/orphanage/old age house/park | 21 | 3.8 |
| Public toilet | 27 | 4.8 |
| Department store / mini market | 39 | 7.0 |
| Stadium/ exercise center/ swimming pool | 39 | 7.0 |
| Tourist hotel | 39 | 7.0 |
| Airport | 4 | 0.7 |
| **Total** | **560** | **100** |

Indicators of Tobacco Law Compliance

Table 2 shows that low use of “no tobacco” signage which was 32% in indoor areas and 13% in outdoor areas but the majority of public places had their signage in visible areas. One in ten public places was observed to be using tobacco products as well as buying and selling tobacco products in indoor areas. Nearly two-fifth (40%) of public places were observed to be using tobacco products in their outdoor area and three-fourth (76%) of public places were observed for buying and selling of tobacco products within their 100m premises. Some of the non-authorized public places had misconceptions and designated their indoor or outdoor area as smoking zones whereas most of the authorized public places had not designated areas as smoking zones. The presence of ashtrays was observed in 8% in both indoor and outdoor areas whereas the presence of lighter/match box was higher in both indoor (13%) and outdoor (22%) areas. The presence of smell of tobacco products was nearly similar to the use of tobacco products but the presence of the remaining of tobacco products was double than observed tobacco use in both indoor and outdoor areas which suggests there is higher tobacco use than observed in public places.

Table 2: Indicators of law compliance in public places (N=560)

| **Indicators** | **Indoor (%)** | **Outdoor (%)** |
| --- | --- | --- |
| Use of signage | 31.8% | 13.4% |
| Visibility of signage | 30.0% | 12.9% |
| Use of tobacco products | 12.7% | 39.6% |
| Selling and buying of tobacco products | 11.8% | 76.2% |
| Designated area as "smoking zone" |  |  |
| Non-authorized public place with "smoking zone" | 5.2% | 8.3% |
| Authorized public place with "smoking zone" | 23.3% | 9.3% |
| Presence of ashtray | 8.8% | 8.4% |
| Presence of lighter/match box | 12.5% | 21.6% |
| Smell of tobacco products | 17.5% | 36.3% |
| Presence of remaining of tobacco products | 25.9% | 65.0% |

**Tobacco law compliance**

Table 3 showed that majority of the hotels/restaurants (50%), bus stops/ticket counters (41.5%), public toilets (55.6%), and tourist hotels (74.4%) had low indoor tobacco law compliance. Similarly, most of educational institutions/libraries (71.1%), religious places/pilgrimage (76.3%), government offices (66.7%), public vehicles (71.8%), industry (69%), hostels (82.1%), department store/mini market (53.8%), stadium/exercise center/swimming pool (56.4%) and airport (75%) had medium indoor tobacco law compliance. However, most of the health institutions, cinema halls and child care/orphanages/old age houses/parks had high indoor tobacco law compliance with 48.6%, 52.2% and 52.4% respectively. The majority of the public vehicle (76.5%), bus stops/ticket counters (63.4%), cinema halls (52.2), public toilets (77.8%), tourist hotels (71.8%) and airports (50%) had low outdoor tobacco compliance whereas all other places i.e. hotel/restaurant (60.5%), educational institution/library (65.8%), religious places/pilgrimage (71.1%), Government office (73.3%), industry (71.4%), health institution (71.4%), hostel (71.4%), child care/orphanage/old age house/park (71.4%), department store/mini market (53.8%) and stadium/exercise center/swimming pool (61.5%) had medium tobacco law compliance in the outdoor area.

Table 3: Tobacco law compliance (Public place wise)

| **Public places** | **Category** | **Indoor** | | **Outdoor** | |
| --- | --- | --- | --- | --- | --- |
|  |  | **Number** | **Percentage** | **Number** | **Percentage** |
| Hotel/restaurant (n=38) | Low | 19 | 50.0 | 15 | 39.5 |
|  | Medium | 11 | 28.9 | 23 | 60.5 |
|  | High | 8 | 21.1 | 0 | 0.0 |
| Educational institution/ library (n=38) | Low | 0 | 0.0 | 8 | 21.1 |
|  | Medium | 27 | 71.1 | 25 | 65.8 |
|  | High | 11 | 28.9 | 5 | 13.2 |
| Religious places/ pilgrimage (n=38) | Low | 1 | 2.6 | 6 | 15.8 |
|  | Medium | 29 | 76.3 | 27 | 71.1 |
|  | High | 8 | 21.1 | 5 | 13.2 |
| Government office (n=30) | Low | 0 | 0.0 | 5 | 16.7 |
|  | Medium | 20 | 66.7 | 22 | 73.3 |
|  | High | 10 | 33.3 | 3 | 10.0 |
| Public vehicle (n=78) | Low | 0 | 0.0 | 62 | 79.5 |
|  | Medium | 56 | 71.8 | 16 | 20.5 |
|  | High | 22 | 28.2 | 0 | 0.0 |
| Industry (n=42) | Low | 4 | 9.5 | 6 | 14.3 |
|  | Medium | 29 | 69.0 | 30 | 71.4 |
|  | High | 9 | 21.4 | 6 | 14.3 |
| Health institution (n=35) | Low | 1 | 2.9 | 6 | 17.1 |
|  | Medium | 17 | 48.6 | 25 | 71.4 |
|  | High | 17 | 48.6 | 4 | 11.4 |
| Bus stop/ticket counter (n=41) | Low | 17 | 41.5 | 26 | 63.4 |
|  | Medium | 17 | 41.5 | 14 | 34.1 |
|  | High | 7 | 17.1 | 1 | 2.4 |
| Hostel (n=28) | Low | 0 | 0.0 | 5 | 17.9 |
|  | Medium | 23 | 82.1 | 20 | 71.4 |
|  | High | 5 | 17.9 | 3 | 10.7 |
| Cinema hall (n=23) | Low | 0 | 0.0 | 12 | 52.2 |
|  | Medium | 11 | 47.8 | 9 | 39.1 |
|  | High | 12 | 52.2 | 2 | 8.7 |
| Child care/orphanage/ old age house/park (n=21) | Low | 1 | 4.8 | 3 | 14.3 |
|  | Medium | 9 | 42.9 | 15 | 71.4 |
|  | High | 11 | 52.4 | 3 | 14.3 |
| Public toilet (n=27) | Low | 15 | 55.6 | 21 | 77.8 |
|  | Medium | 10 | 37.0 | 6 | 22.2 |
|  | High | 2 | 7.4 | 0 | 0.0 |
| Department store / mini market (n=39) | Low | 0 | 0.0 | 12 | 30.8 |
|  | Medium | 21 | 53.8 | 21 | 53.8 |
|  | High | 18 | 46.2 | 6 | 15.4 |
| Stadium/ exercise center/ swimming pool (n=39) | Low | 6 | 15.4 | 11 | 28.2 |
|  | Medium | 22 | 56.4 | 24 | 61.5 |
|  | High | 11 | 28.2 | 4 | 10.3 |
| Tourist hotel (n=39) | Low | 29 | 74.4 | 28 | 71.8 |
|  | Medium | 9 | 23.1 | 10 | 25.6 |
|  | High | 1 | 2.6 | 1 | 2.6 |
| Airport (n=4) | Low | 1 | 25.0 | 2 | 50.0 |
|  | Medium | 3 | 75.0 | 2 | 50.0 |
|  | High | 0 | 0.0 | 0 | 0.0 |

Table 4 shows that majority public places of Koshi province, Gandaki province, Lumbini province and Sudurpashchim province have medium level of tobacco law compliance in both indoor and outdoor areas. Majority public places of Bagmati and Karnali province have medium level of indoor tobacco law compliance but low level of outdoor law compliance. Madesh province has high level of indoor tobacco law compliance but outdoor law compliance is low.

Table 4: Tobacco law compliance (Province wise)

| **Province** | **Category** | **Indoor** | | **Outdoor** | |
| --- | --- | --- | --- | --- | --- |
|  |  | **Number** | **Percentage** | **Number** | **Percentage** |
| Koshi Province  (Biratnagar) | Low | 9 | 11.3 | 27 | 33.8 |
|  | Medium | 52 | 65.0 | 47 | 58.8 |
|  | High | 19 | 23.7 | 6 | 7.5 |
| Madesh Province (Janakpur) | Low | 18 | 22.5 | 45 | 56.3 |
|  | Medium | 25 | 31.3 | 32 | 40.0 |
|  | High | 37 | 46.2 | 3 | 3.8 |
| Bagmati Province (Hetauda) | Low | 21 | 26.3 | 46 | 57.5 |
|  | Medium | 41 | 51.2 | 27 | 33.8 |
|  | High | 18 | 22.5 | 7 | 8.8 |
| Gandaki Province (Pokhara) | Low | 12 | 15.0 | 17 | 21.3 |
|  | Medium | 47 | 58.7 | 56 | 70.0 |
|  | High | 21 | 26.3 | 7 | 8.8 |
| Lumbini Province (Butwal) | Low | 12 | 15.0 | 29 | 36.3 |
|  | Medium | 47 | 58.7 | 36 | 45.0 |
|  | High | 21 | 26.3 | 15 | 18.8 |
| Karnali Province (Surkhet) | Low | 14 | 17.5 | 47 | 58.8 |
|  | Medium | 45 | 56.2 | 32 | 40.0 |
|  | High | 21 | 26.3 | 1 | 1.4 |
| Sudurpaschim Province (Dhangadi) | Low | 8 | 10.0 | 17 | 21.3 |
|  | Medium | 57 | 71.2 | 59 | 73.8 |
|  | High | 15 | 18.8 | 4 | 5.0 |

**DISCUSSION**

The assessment of the compliance status with the smoke-free public places law in Nepal reveals several noteworthy observations that warrant discussion. The study brings attention to the limited presence of no tobacco signage in public places in Nepal which was a similar to study in Pakistan which may be due to the limited supply of no tobacco signage and less practice of fines for non-compliance with signage. In contrast, studies in India and Turkey demonstrate a higher prevalence of "no tobacco" signage, suggesting potential better enforcement of law and regulatory mechanisms [[15-18](#_ENREF_15)]. Noteworthy is the provision within the Tobacco Product (Control and Regulatory) Act, imposing a substantial fine of 5000 NPR for non-compliance with signage placement in office spaces. The imposition of such a stringent penalty underscores the gravity of this issue for policymakers and program implementers, despite observed low compliance in public places.

Despite regulatory endeavors, a sustained prevalence of tobacco use in public areas is evident. Tobacco use and its buying and selling were low in indoor areas as compared to outdoor areas and a similar result was seen in studies from India and Turkey [[18](#_ENREF_18), [19](#_ENREF_19)]. The presence of the smell of tobacco products was nearly similar to the use of tobacco products but the presence of the remaining of tobacco products was double than observed use of tobacco. Similar findings were seen in studies from India and Turkey which suggested that there was higher tobacco use than observed in public places [[18](#_ENREF_18), [20](#_ENREF_20), [21](#_ENREF_21)]. The pronounced frequency of tobacco use in outdoor spaces necessitates the strict implementation of law along with targeted interventions and intensified awareness initiatives to redress this facet of non-compliance.

The majority of hotels/restaurants, bus stops/ticket counters, public toilets, and tourist hotels exhibited low compliance with indoor tobacco laws. On the contrary, health institutions, cinema halls, and child care/old age houses demonstrated a higher level of indoor tobacco law compliance. This discrepancy in compliance rates could be influenced by various factors. Hotels/restaurants, bus stops, and public toilets being public spaces with diverse foot traffic, may face challenges in enforcing indoor smoking regulations due to the transient nature of visitors. In contrast, health institutions, cinema halls, and child care/old age houses may have stricter enforcement measures or a more health-conscious environment, contributing to higher compliance rates.

Similarly, the majority of the public vehicles, bus stops/ticket counters, cinema halls, public toilets, and tourist hotels had low outdoor tobacco compliance although one of these public places had high indoor tobacco law compliance. These findings were similar to the study done in India and Turkey [[16](#_ENREF_16), [18](#_ENREF_18), [21](#_ENREF_21), [22](#_ENREF_22)]. This low outdoor compliance rate could be influenced by various factors such as enforcement practices and public awareness. Public vehicles, bus stops, and tourist hotels may face challenges in ensuring outdoor tobacco compliance due to the open and transient nature of these spaces. Further research to identify the specific factors contributing to low outdoor compliance and outliers can guide targeted interventions to address these challenges effectively in the future. Furthermore, recognizing the outlier with high indoor tobacco law compliance among outdoor public places presents an opportunity to investigate and replicate successful strategies in other similar settings. Implementation of the Tobacco Product (Control and Regulatory) Act has brought some improvement in tobacco smoke free public places like indoor areas of health institutions, cinema halls, child care/old age houses, and public vehicles. Tobacco use and exposure to secondhand smoke have decreased in 2019 as compared to 2013[[5](#_ENREF_5)] which can be related to the implementation of the Tobacco Product (Control and Regulatory) Act. But public areas like hotels (including tourist hotels)/restaurants, bus stops/ticket counters, public toilets, and outdoor areas of public vehicles and cinema halls still needs strict enforcement of law.

A similar pattern across the provinces in indoor tobacco law compliance was seen but Madesh, Bagmati, and Karnali provinces had lower outdoor law compliance than the other four provinces. This discrepancy in outdoor compliance could stem from various factors, including differences in enforcement practices, public awareness, cultural norms, or regional variations in the understanding and acceptance of tobacco control measures. So, strategies to improve compliance, particularly in outdoor settings, may need to be region-specific to address the unique challenges faced by the Madhes, Bagmati, and Karnali provinces. Nepal NCD STEPS survey 2019 showed Koshi, Bagmati and Gandaki provinces had tobacco use below the national average and the other four provinces had high tobacco use [[5](#_ENREF_5)]. These findings suggested Koshi and Gandaki provinces had medium tobacco law compliance and low tobacco use whereas Madesh and Karnali provinces had low outdoor law compliance and high tobacco use which are coherent findings. Although Bagamti province had low outdoor law compliance, tobacco use was low. Similarly, Lumbini and Sudurpaschim province had medium law compliance but tobacco use was high. This variation may be due to differences in education and socio-economic status among provinces.

This study is one of a kind that explores tobacco law compliance across the country. This study examines the compliance status of smoke free public place law in the provincial headquarters of Nepal which are urban settings so these findings can be generalized to similar urban settings. But rural settings of Nepal may have different smoke free public place law compliance so further study is needed to explore law compliance in rural settings.

# **CONCLUSION**

The assessment of the compliance status with the smoke-free public places law in Nepal reveals key insights into the current state of tobacco control measures. It is evident that while progress has been made in certain aspects of tobacco law compliance, still there remains a compelling need for comprehensive and targeted interventions to ensure adherence to laws in public places across the country. The findings of this study provide valuable insights for policymakers and enforcement agencies to develop and implement effective strategies to create tobacco-free environments in public places.

**Funding**

This study was funded by the Government of Nepal.

**Disclosure**

The funders had no role in the study design, data collection, analysis, and manuscript preparation.

**Conflicts of Interest**

The authors declare no conflicts of interest.

**Authors’ Contributions**

AS and BBK contributed to conceptualization, project administration, reviewing, and editing. RS contributed to the analysis and writing of the original draft. All authors read and approved the manuscript.

# **Supplementary files**

1. Table 1: Types of public places
2. Table 2: Indicators of law compliance in public places
3. Table 3: Tobacco law compliance (Public place wise)
4. Table 4: Tobacco law compliance (Province wise)
5. English questionnaire

# **REFERENCES**

1. Organization WH. WHO report on the global tobacco epidemic, 2008: the MPOWER package: World Health Organization; 2008.

2. Organization WH. WHO report on the global tobacco epidemic, 2023: protect people from tobacco smoke. 2023.

3. Brown JL, Smith KC, Welding K, Barnoya J, Cohen JE. Innovations that harm: tobacco product and packaging in low-income and middle-income countries. BMJ Specialist Journals; 2023.

4. Nepal Go. Multisectoral action plan for the prevention and control of non communicable diseases (2014–2020). Government of Nepal Kathmandu; 2014.

5. Dhimal M BB, Bhattarai S, Dixit LP, Hyder MKA, Agrawal N, Rani M, Jha AK. Report of Non Communicable Disease Risk Factors: STEPS Survey Nepal 2019. Kathmandu: Nepal Health Research Council, 2020.

6. BB K. Nepal Global Youth Tobacco Survey (GYTS) FACT SHEET. 2011.

7. Kabir M, Goh K-L. Determinants of tobacco use among students aged 13–15 years in Nepal and Sri Lanka: results from the Global Youth Tobacco Survey, 2007. Health Education Journal. 2014;73(1):51-61.

8. Karki K, Dahal B, Regmi A, Poudel A, Gurung Y. WHO STEPS Surveillance: Non Communicable Diseases Risk Factors Survey. 2008. Kathmandu: Ministry of Health and Population, GoN, Society for Local Integrated Development Nepal (SOLID Nepal) and WHO. 2008.

9. Bhakta K, Oli LK, Dahal N. Awareness and Implementation Status of Tobacco Policy Provisions among Students of Public Schools in Budhanilkantha Municipality, Nepal. Journal of Karnali Academy of Health Sciences. 2020;3(2):41-6.

10. Kunugita N. The SDGS and tobacco measures under the World Health Organization Framework Convention on Tobacco Control (FCTC). Journal of the National Institute of Public Health. 2019;68(5):387-94.

11. Dahal S, Subedi R, Maharjan S, Maharjan J. Smoking behavior of adolescents and their view towards government's ban on smoking in public places in Kathmandu. Nepal Journal of Medical Sciences. 2014;3(2):94-100.

12. Pradhan PMS, Niraula SR, Ghimire A, Singh SB, Pokharel PK. Tobacco use and associated factors among adolescent students in Dharan, Eastern Nepal: a cross-sectional questionnaire survey. BMJ open. 2013;3(2):e002123.

13. Aryal UR, Petzold M, Krettek A. Perceived risks and benefits of cigarette smoking among Nepalese adolescents: a population-based cross-sectional study. BMC public health. 2013;13(1):1-9.

14. Sreeramareddy CT, Kishore P, Paudel J, Menezes RG. Prevalence and correlates of tobacco use amongst junior collegiates in twin cities of western Nepal: a cross-sectional, questionnaire-based survey. BMC Public Health. 2008;8(1):1-8.

15. Khan JA, Amir Humza Sohail AM, Arif Maan MA. Tobacco control laws in Pakistan and their implementation: A pilot study in Karachi. Journal of Pakistan Medical Association. 2016;66(7):875.

16. Goel S, Ravindra K, Singh RJ, Sharma D. Effective smoke-free policies in achieving a high level of compliance with smoke-free law: experiences from a district of North India. Tobacco control. 2014;23(4):291-4.

17. Jain M, Chauhan M, Singh R. Compliance assessment of cigarette and other tobacco products act in public places of Alwar district of Rajasthan. Indian Journal of Public Health. 2016;60(2):107.

18. Navas-Acien A, Çarkoğlu A, Ergör G, Hayran M, Ergüder T, Kaplan B, et al. Compliance with smoke-free legislation within public buildings: a cross-sectional study in Turkey. Bulletin of the World Health Organization. 2016;94(2):92.

19. Rijhwani K, Mohanty VR, Balappanavar AY, Hashmi S. Compliance assessment of cigarette and other tobacco products act in public places in Delhi government hospitals. Asian Pacific Journal of Cancer Prevention: APJCP. 2018;19(8):2097.

20. Kaplan B, Grau-Perez M, Çarkoglu A, Ergör G, Hayran M, Navas-Acien A, et al. Smoke-free Turkey: Evaluation of outdoor areas of public places. Environmental research. 2019;175:79-83.

21. Kumar R, Chauhan G, Satyanarayana S, Lal P, Singh RJ, Wilson NC. Assessing compliance to smoke-free legislation: results of a sub-national survey in Himachal Pradesh, India. WHO South-East Asia Journal of Public Health. 2013;2(1):52-6.

22. Goel S, Sardana M, Jain N, Bakshi D. Descriptive evaluation of cigarettes and other tobacco products act in a North Indian city. Indian Journal of Public Health. 2016;60(4):273.
