# Supplementary material for: Changes in the Biomechanical Properties of Corneal Stromal Lens after Collagen Crosslinking Induced by EDC-NHS
Source: J Ophthalmol. 2024 May 17;2024:9943458. doi: 10.1155/2024/9943458 (PMC11126343; doi:10.1155/2024/9943458)
Supplement: Supplementary Materials — In this study, the preservation methods of corneal stromal lens, collagen crosslinking methods, and inflation testing were based on the previous research results of our team [16] and the research results of Matthew et al. [17]. [file 9943458.f1.zip › Enzymatic degradation experiments..pdf]

0.0046	0.0041	0.0030	0.0025	0.0028
0.0044	0.0030	0.0028	0.0024	0.0029
0.0032	0.0045	0.0025	0.0038	0.0027
0.0026	0.0046	0.0026	0.0042	0.0029
0.0034	0.0039	0.0039	0.0054	0.0039
0.0041	0.0039	0.0040	0.0058	0.0041

0.0039	0.0031	0.0026	0.0021	0.0022
0.0041	0.0026	0.0022	0.0026	0.0025
0.0030	0.0032	0.0021	0.0029	0.0020
0.0026	0.0033	0.0025	0.0035	0.0025
0.0032	0.0029	0.0038	0.0046	0.0035
0.0044	0.0030	0.0038	0.0050	0.0043

0.0037		0.0026	0.0021	0.0031
0.0025	0.0019	0.0022	0.0025	0.0042
0.0032	0.0029	0.0019	0.0030	0.0023
0.0027	0.0027	0.0023	0.0040	
0.0037	0.0027	0.0036	0.0046	0.0037
0.0041	0.0027	0.0034	0.0050	0.0050

		0.0026	0.0017	0.0029
	0.0023	0.0028	0.0027	
0.0032	0.0025	0.0026	0.0034	
0.0025		0.0028	0.0035	
	0.0028	0.0036	0.0039	0.0026
0.0028		0.0036	0.0043	0.0034

		0.0023	0.0013	
		0.0024	0.0024	
	0.0014	0.0020	0.0024	
		0.0021	0.0025	
	0.0015	0.0038	0.0034	
		0.0038	0.0034	

			0.0012	
		0.0019	0.0022	
		0.0023	0.0020	
			0.0019	
		0.0035	0.0028	
		0.0033		

			0.0009	
			0.0018	
		0.0034		
		0.0033		

			0.0002	
			0.0018	
		0.0024		

0.0031

0.0018

0.0024

0.0018

0.0029	0.0028	0.0034	0.0028	0.0029
0.0032	0.0029	0.0047	0.0029	0.0027
0.0058	0.0029	0.0033	0.0033	0.0044
0.0052	0.0038	0.0029	0.0029	0.0022
0.0059	0.0035	0.0035	0.0027	0.0046
**0.023**	**0.0159**	**0.0178**	**0.0146**	**0.0168**
0.0034	0.0027	0.0029	0.0028	0.0018
0.0034	0.0032	0.0038	0.0022	0.0020
0.0050	0.0024	0.0021	0.0029	0.0039
0.0039	0.0035	0.0021	0.0030	0.0019
0.0041	0.0022	0.0026	0.0027	0.0036
**0.0198**	**0.014**	**0.0135**	**0.0136**	**0.0132**
0.0028	0.0030	0.0021	0.0023	0.0024
0.0025	0.0032	0.0032	0.0027	0.0019
0.0045	0.0020	0.0020	0.0025	0.0034
0.0038	0.0029	0.0018	0.0027	0.0018
0.0045	0.0021	0.0027	0.0025	0.0034
**0.0181**	**0.0132**	**0.0118**	**0.0127**	**0.0129**
0.0021	0.0023	0.0021	0.0023	0.0020
0.0007	0.0024	0.0030	0.0015	0.0019
0.0024	0.0014	0.0021	0.0026	0.0033
0.0032	0.0023	0.0013	0.0021	0.0016
0.0026	0.0015	0.0019	0.0020	0.0028
**0.011**	**0.0099**	**0.0104**	**0.0105**	**0.0116**
	0.0019	0.0020	0.0022	0.0020
	0.0023	0.0026	0.0008	0.0018
	0.0004	0.0014	0.0022	0.0031
	0.0017	0.0018	0.0019	0.0018
	0.0013	0.0022	0.0017	0.0021
	**0.0076**	**0.01**	**0.0088**	**0.0108**
	0.0016	0.0014	0.0016	
	0.0023	0.0030	0.0004	
	0.0005	0.0016	0.0024	
	0.0018		0.0015	
	0.0007	0.0022	0.0019	
	**0.0069**	**0.0082**	**0.0078**	
	0.0013		0.0014	
	0.0018	0.0030	0.0002	
	0.0002	0.0013	0.0018	
	0.0001	0.0016	0.0018	
		0.0024	0.0011	
	**0.0034**	**0.0083**	**0.0063**	
	0.0010		0.0011	
	0.0016	0.0011	0	
			0.0015	
		0.0013	0.0016	
			0.0008	
	**0.0026**	**0.0024**	**0.005**	
			0.0005	
	0.0011			
			0.0010	
		0.0010	0.0014	
			0.0008	
	**0.0011**	**0.001**	**0.0037**	

		0.0001
0.0012		
		0.0012
		0.0012
		0.0004
**0.0012**	**0**	**0.0029**

Time(h)	Total weig	Residual	Group
0	0.023	5	Control
1	0.0198	5	Control
2	0.0181	5	Control
3	0.011	5	Control
4	0		Control
0	0.0159	5	5/2.5
1	0.014	5	5/2.5
2	0.0132	5	5/2.5
3	0.0099	5	5/2.5
4	0.0076	5	5/2.5
5	0.0069	5	5/2.5
6	0.0034	4	5/2.5
7	0.0026	2	5/2.5
8	0.0011	1	5/2.5
9	0.0012	1	5/2.5
0	0.0178	5	5/5
1	0.0135	5	5/5
2	0.0118	5	5/5
3	0.0104	5	5/5
4	0.01	5	5/5
5	0.0082	4	5/5
6	0.0083	4	5/5
7	0.0024	2	5/5
8	0.001	1	5/5
9	0		5/5
0	0.0146	5	10/5
1	0.0136	5	10/5
2	0.0127	5	10/5
3	0.0105	5	10/5
4	0.0088	5	10/5
5	0.0078	5	10/5
6	0.0063	5	10/5
7	0.005	4	10/5
8	0.0037	4	10/5
9	0.0029	4	10/5
0	0.0168	5	UVA
1	0.0132	5	UVA
2	0.0129	5	UVA
3	0.0116	5	UVA
4	0.0108	5	UVA
5	0		UVA

Time(h)	Residual	Group
0	5	Control
1	5	Control
2	5	Control
3	5	Control
0	5	5/2.5
1	5	5/2.5
2	5	5/2.5
3	5	5/2.5
4	5	5/2.5
5	5	5/2.5
6	4	5/2.5
7	2	5/2.5
8	1	5/2.5
9	1	5/2.5
0	5	5/5
1	5	5/5
2	5	5/5
3	5	5/5
4	5	5/5
5	4	5/5
6	4	5/5
7	2	5/5
8	1	5/5
0	5	10/5
1	5	10/5
2	5	10/5
3	5	10/5
4	5	10/5
5	5	10/5
6	5	10/5
7	4	10/5
8	4	10/5
9	4	10/5
0	5	B2
1	5	B2
2	5	B2
3	5	B2
4	5	B2

Time(h)	Total wei	Residual	Group
0	0.0223	6	Control
1	0.0212	6	Control
2	0.0199	6	Control
3	0.0085	3	Control
4	0		Control
0	0.024	6	5/2.5
1	0.0181	6	5/2.5
2	0.0129	5	5/2.5
3	0.0076	3	5/2.5
4	0.0029	2	5/2.5
5	0		5/2.5
0	0.0188	6	5/5
1	0.017	6	5/5
2	0.016	6	5/5
3	0.018	6	5/5
4	0.0164	6	5/5
5	0.011	4	5/5
6	0.0067	2	5/5
7	0.0055	2	5/5
8	0.0024	1	5/5
9	0		5/5
0	0.0241	6	10/5
1	0.0207	6	10/5
2	0.0212	6	10/5
3	0.0195	6	10/5
4	0.0154	6	10/5
5	0.0101	5	10/5
6	0.0027	2	10/5
7	0.002	2	10/5
8	0.0018	1	10/5
9	0.0018	1	10/5
0	0.0193	6	UVA
1	0.017	6	UVA
2	0.0183	5	UVA
3	0.0089	3	UVA
4	0		UVA
