# Supplementary material for: Changes in the Biomechanical Properties of Corneal Stromal Lens after Collagen Crosslinking Induced by EDC-NHS
Source: J Ophthalmol. 2024 May 17;2024:9943458. doi: 10.1155/2024/9943458 (PMC11126343; doi:10.1155/2024/9943458)
Supplement: Supplementary Materials — In this study, the preservation methods of corneal stromal lens, collagen crosslinking methods, and inflation testing were based on the previous research results of our team [16] and the research results of Matthew et al. [17]. [file 9943458.f1.zip › Experimental data of lens edema detected by wavelength scanning.pdf]

WL(nm)	CS1	CS2	CS3	CS4	CS5	Con	cxl11	cxl12
780	88.9	89.2	85.2	87	87.6	87.58	89.9	92.6
775	89.2	89.2	85.4	87	87.7	87.7	90	92.7
770	89.3	89.2	85.3	87	87.5	87.66	90	92.8
765	89.3	89.2	85.3	87	87.5	87.66	90.1	92.8
760	89.3	89.2	85.2	86.9	87.5	87.62	90.1	92.7
755	89.2	89.1	85.1	86.7	87.5	87.52	90	92.7
750	89.1	88.9	85	86.6	87.3	87.38	89.8	92.7
745	89	88.9	84.8	86.5	87.1	87.26	89.8	92.6
740	88.9	88.8	84.7	86.4	87	87.16	89.8	92.6
735	88.7	88.6	84.6	86.3	86.9	87.02	89.7	92.6
730	88.6	88.5	84.4	86.1	86.7	86.86	89.6	92.5
725	88.5	88.4	84.2	86	86.6	86.74	89.5	92.5
720	88.5	88.4	84.1	85.9	86.6	86.7	89.4	92.5
715	88.4	88.3	84	85.8	86.4	86.58	89.3	92.4
710	88.3	88.1	83.8	85.6	86.2	86.4	89.2	92.3
705	88.2	87.9	83.6	85.5	86.1	86.26	89.2	92.3
700	88.1	87.8	83.5	85.3	86.1	86.16	89.1	92.3
695	87.9	87.7	83.4	85.2	85.9	86.02	89.1	92.2
690	87.8	87.5	83.2	85	85.7	85.84	89	92.2
685	87.7	87.5	83	84.9	85.6	85.74	88.9	92.1
680	87.5	87.3	82.8	84.7	85.4	85.54	88.8	92
675	87.3	87.1	82.6	84.5	85.3	85.36	88.6	92
670	87.3	87	82.6	84.5	85.1	85.3	88.6	92
665	87.3	86.9	82.5	84.4	85.1	85.24	88.6	92
660	87.1	86.8	82.2	84.2	84.9	85.04	88.4	91.9
655	87	86.6	82	84	84.7	84.86	88.3	91.8
650	86.8	86.4	81.9	83.8	84.5	84.68	88.2	91.7
645	86.7	86.3	81.7	83.7	84.3	84.54	88.2	91.7
640	86.6	86.2	81.6	83.5	84.2	84.42	88	91.6
635	86.5	86.1	81.3	83.4	84	84.26	87.9	91.6
630	86.3	85.9	81.2	83.2	83.9	84.1	87.9	91.6
625	86.2	85.8	81	83.1	83.7	83.96	87.8	91.5
620	86.1	85.6	80.9	83	83.4	83.8	87.6	91.5
615	85.9	85.4	80.7	82.9	83.2	83.62	87.5	91.4
610	85.7	85.2	80.4	82.6	83	83.38	87.3	91.3
605	85.5	84.9	80.2	82.3	82.7	83.12	87.1	91.1
600	85.3	84.6	79.9	82.1	82.5	82.88	87	91
595	85.2	84.5	79.8	81.9	82.3	82.74	86.9	91.1
590	85.1	84.3	79.6	81.8	82.2	82.6	86.9	91
585	84.9	84.1	79.4	81.6	81.9	82.38	86.7	91
580	84.7	83.8	79.2	81.4	81.7	82.16	86.6	90.9
575	84.5	83.7	79	81.2	81.5	81.98	86.5	90.8
570	84.3	83.4	78.7	81	81.3	81.74	86.3	90.7
565	84.1	83.1	78.4	80.8	81.1	81.5	86.2	90.7
560	83.9	82.9	78.2	80.6	80.8	81.28	86.1	90.6
555	83.7	82.6	77.9	80.3	80.5	81	85.9	90.5
550	83.4	82.2	77.6	80	80.3	80.7	85.8	90.5
545	83.1	81.9	77.3	79.7	80	80.4	85.6	90.4
540	82.9	81.7	77	79.4	79.8	80.16	85.4	90.3
535	82.7	81.5	76.7	79.1	79.5	79.9	85.3	90.2
530	82.5	81.2	76.4	78.9	79.2	79.64	85.1	90.2
525	82.2	80.9	76.1	78.6	78.9	79.34	84.9	90
520	82	80.6	75.8	78.3	78.6	79.06	84.8	89.9

515	81.7	80.3	75.5	78	78.3	78.76	84.6	89.8
510	81.5	80	75.2	77.7	78	78.48	84.4	89.7
505	81.2	79.7	74.8	77.4	77.7	78.16	84.3	89.7
500	80.9	79.4	74.4	77	77.3	77.8	84.1	89.6
495	80.6	79	74	76.7	77	77.46	83.9	89.5
490	80.3	78.7	73.6	76.3	76.7	77.12	83.6	89.3
485	80	78.4	73.2	76	76.3	76.78	83.4	89.2
480	79.6	77.9	72.8	75.6	75.8	76.34	83.2	89.1
475	79.2	77.5	72.4	75.2	75.4	75.94	83	88.9
470	78.8	77.1	72	74.8	74.9	75.52	82.7	88.8
465	78.5	76.7	71.6	74.3	74.5	75.12	82.5	88.6
460	78.1	76.2	71.1	73.8	74.2	74.68	82.2	88.5
455	77.7	75.7	70.6	73.4	73.7	74.22	81.9	88.4
450	77.3	75.2	70.1	72.9	73.2	73.74	81.6	88.2
445	76.8	74.7	69.5	72.3	72.7	73.2	81.3	88.1
440	76.4	74.2	69	71.8	72.2	72.72	81	87.9
435	76	73.7	68.5	71.4	71.6	72.24	80.8	87.7
430	75.5	73.1	67.9	70.8	71	71.66	80.5	87.5
425	75	72.5	67.3	70.2	70.5	71.1	80.1	87.3
420	74.4	71.9	66.7	69.5	69.9	70.48	79.7	87.1
415	73.9	71.3	66.1	69	69.2	69.9	79.4	86.9
410	73.2	70.6	65.4	68.5	68.5	69.24	79.1	86.8
405	72.6	70	64.7	67.9	67.9	68.62	78.7	86.6
400	72	69.4	64	67.3	67.3	68	78.3	86.4
395	71.4	68.6	63.3	66.6	66.5	67.28	77.9	86.3
390	70.7	67.8	62.6	65.9	65.7	66.54	77.5	86
385	70.1	67	61.8	65.2	64.9	65.8	77.2	85.7
380	69.6	66.4	61.2	64.7	64.3	65.24	76.9	85.5

cxl13	cxl14	cxl15	5/2.5	cxl21	cxl22	cxl23	cxl24	cxl25
92.9	92.1	90.8	91.66	91.7	92.2	91.7	93.3	90.3
93.1	92.2	91	91.8	91.9	92.3	91.8	93.5	90.4
93.2	92.2	91	91.84	92	92.3	91.8	93.5	90.5
93.3	92.2	91.1	91.9	92	92.2	91.8	93.5	90.5
93.3	92.1	91	91.84	91.9	92.1	91.7	93.5	90.4
93.2	92.1	91	91.8	91.7	92	91.7	93.4	90.3
93.1	92.1	90.9	91.72	91.7	91.9	91.6	93.4	90.3
93	92	90.9	91.66	91.7	91.9	91.6	93.4	90.2
93	91.9	90.8	91.62	91.6	91.7	91.5	93.4	90.1
92.9	91.9	90.7	91.56	91.4	91.6	91.4	93.3	90.1
92.9	91.8	90.6	91.48	91.5	91.6	91.3	93.2	90
92.9	91.8	90.6	91.46	91.5	91.6	91.3	93.2	89.9
92.9	91.7	90.6	91.42	91.4	91.5	91.3	93.1	89.8
92.8	91.6	90.6	91.34	91.2	91.4	91.2	93	89.8
92.7	91.5	90.4	91.22	91.2	91.3	91	92.9	89.7
92.6	91.4	90.4	91.18	91.1	91.1	91	92.8	89.6
92.6	91.4	90.3	91.14	91	91	90.9	92.8	89.5
92.5	91.4	90.3	91.1	90.8	90.8	90.8	92.7	89.5
92.4	91.3	90.2	91.02	90.7	90.7	90.7	92.6	89.4
92.4	91.2	90.1	90.94	90.6	90.6	90.6	92.5	89.4
92.3	91.1	90	90.84	90.5	90.5	90.5	92.4	89.2
92.2	91	89.9	90.74	90.4	90.3	90.4	92.3	89.1
92.2	91	89.9	90.74	90.4	90.3	90.4	92.3	89.1
92.2	91	89.9	90.74	90.3	90.2	90.3	92.3	89
92	90.8	89.9	90.6	90.2	90.1	90.2	92.2	88.9
92	90.7	89.8	90.52	90.1	90	90.1	92	88.8
91.9	90.6	89.7	90.42	90.1	89.9	90	92	88.7
91.8	90.6	89.6	90.38	90	89.7	89.9	91.9	88.6
91.8	90.5	89.5	90.28	89.9	89.6	89.8	91.8	88.5
91.7	90.4	89.4	90.2	89.7	89.5	89.7	91.8	88.4
91.7	90.3	89.4	90.18	89.6	89.3	89.6	91.7	88.3
91.7	90.2	89.3	90.1	89.5	89.1	89.6	91.6	88.3
91.6	90	89.2	89.98	89.4	89	89.4	91.5	88.2
91.6	89.9	89.1	89.9	89.3	88.9	89.3	91.4	88.1
91.5	89.9	89	89.8	89	88.7	89.2	91.3	87.9
91.3	89.7	88.9	89.62	88.8	88.4	89	91.1	87.7
91.2	89.5	88.8	89.5	88.7	88.2	88.8	90.9	87.5
91.1	89.5	88.7	89.46	88.6	88.1	88.7	90.8	87.5
91.1	89.4	88.7	89.42	88.5	87.9	88.6	90.8	87.5
91	89.3	88.6	89.32	88.4	87.7	88.5	90.7	87.4
90.9	89.2	88.5	89.22	88.2	87.5	88.4	90.5	87.3
90.8	89.1	88.5	89.14	88	87.3	88.2	90.4	87.1
90.7	89	88.3	89	87.9	87.1	88.1	90.2	87
90.6	88.9	88.2	88.92	87.7	86.9	88	90.1	86.9
90.5	88.8	88.1	88.82	87.6	86.7	87.8	90	86.8
90.3	88.6	88	88.66	87.5	86.5	87.6	89.8	86.6
90.2	88.4	87.8	88.54	87.3	86.3	87.4	89.6	86.4
90.1	88.3	87.7	88.42	87.1	86	87.3	89.4	86.2
90	88.1	87.6	88.28	86.8	85.8	87.1	89.3	86
89.9	88	87.5	88.18	86.7	85.6	87	89.2	86
89.7	87.9	87.4	88.06	86.5	85.4	86.8	89.1	85.8
89.6	87.8	87.3	87.92	86.3	85	86.7	88.8	85.6
89.5	87.6	87.1	87.78	86.1	84.7	86.5	88.6	85.4

89.3	87.4	87	87.62	85.9	84.5	86.2	88.4	85.2
89.2	87.2	86.9	87.48	85.7	84.3	86	88.2	85.1
89.1	87.1	86.8	87.4	85.5	84	85.8	88.1	85
89	86.9	86.6	87.24	85.2	83.6	85.6	87.9	84.8
88.8	86.7	86.4	87.06	85	83.3	85.4	87.6	84.6
88.6	86.5	86.3	86.86	84.7	83	85.2	87.4	84.4
88.4	86.3	86.2	86.7	84.5	82.7	85	87.2	84.2
88.2	86.1	85.9	86.5	84.2	82.3	84.7	86.9	83.9
88.1	85.9	85.8	86.34	83.9	81.9	84.4	86.6	83.7
87.9	85.6	85.6	86.12	83.7	81.5	84.2	86.3	83.4
87.7	85.4	85.4	85.92	83.3	81.1	84	86.1	83.1
87.6	85.2	85.2	85.74	83	80.7	83.7	85.8	82.8
87.3	85	85	85.52	82.7	80.3	83.3	85.5	82.5
87.1	84.7	84.8	85.28	82.3	79.8	83	85.2	82.2
86.9	84.4	84.6	85.06	82	79.4	82.7	84.8	81.8
86.7	84.1	84.4	84.82	81.6	78.9	82.3	84.5	81.5
86.5	83.8	84.1	84.58	81.3	78.4	82	84.2	81.3
86.2	83.6	83.9	84.34	80.9	77.8	81.7	83.8	81
86	83.3	83.6	84.06	80.4	77.3	81.3	83.4	80.5
85.6	82.9	83.3	83.72	80	76.7	80.9	83	80
85.3	82.5	82.9	83.4	79.6	76	80.4	82.5	79.7
85.1	82.1	82.7	83.16	79.1	75.4	80	82.1	79.4
84.8	81.9	82.5	82.9	78.7	74.8	79.7	81.7	79.1
84.5	81.5	82.1	82.56	78.2	74.1	79.2	81.2	78.8
84.2	81.1	81.8	82.26	77.7	73.3	78.7	80.7	78.4
83.9	80.6	81.4	81.88	77.2	72.6	78.1	80.1	78
83.6	80.2	81.1	81.56	76.6	71.8	77.6	79.6	77.6
83.3	79.8	80.9	81.28	76.2	71.3	77.2	79.1	77.3

5/5.0	cxl31	cxl32	cxl33	cxl34	cxl35	10/5.0	B21	B22
91.84	91.3	84.5	91.8	91.9	91.7	90.24	91.3	95.6
91.98	91.3	84.5	91.9	92.1	91.8	90.32	91.4	95.9
92.02	91.4	84.5	92	92.2	91.9	90.4	91.3	96.2
92	91.3	84.5	92.1	92.3	92	90.44	91.2	96.1
91.92	91.2	84.4	92	92.2	91.9	90.34	91.5	95.7
91.82	91.1	84.3	91.7	92	91.8	90.18	91.5	95.4
91.78	91	84.2	91.7	92	91.7	90.12	91.4	95.5
91.76	90.9	84.1	91.7	92	91.6	90.06	91.3	95.4
91.66	90.9	84.1	91.6	91.9	91.5	90	91.2	95.3
91.56	91	84.1	91.6	91.9	91.4	90	91.3	95.1
91.52	90.9	84.1	91.6	91.9	91.5	90	91.4	94.9
91.5	90.7	84	91.7	91.8	91.4	89.92	91.3	94.9
91.42	90.7	84	91.6	91.7	91.4	89.88	91.1	95
91.32	90.6	83.9	91.5	91.6	91.3	89.78	90.8	94.9
91.22	90.5	83.8	91.4	91.6	91.3	89.72	90.6	94.7
91.12	90.4	83.8	91.3	91.6	91.2	89.66	90.5	94.5
91.04	90.3	83.7	91.2	91.4	91	89.52	90.5	94.4
90.92	90.2	83.6	91.1	91.3	90.9	89.42	90.5	94.3
90.82	90.1	83.5	91	91.2	90.8	89.32	90.3	94.1
90.74	90.1	83.3	91	91.1	90.7	89.24	89.8	94
90.62	90	83.1	90.9	91	90.6	89.12	89.9	94.1
90.5	89.9	83.1	90.8	90.9	90.5	89.04	90.1	93.9
90.5	89.8	83.2	90.8	90.9	90.5	89.04	90.1	93.5
90.42	89.8	83.1	90.8	90.8	90.4	88.98	90.2	93.5
90.32	89.7	83	90.7	90.7	90.3	88.88	90.3	93.8
90.2	89.6	82.8	90.6	90.7	90.2	88.78	89.9	93.6
90.14	89.5	82.8	90.6	90.7	90.1	88.74	89.7	93.3
90.02	89.4	82.6	90.5	90.5	90.1	88.62	89.8	93.2
89.92	89.3	82.5	90.4	90.4	90	88.52	89.7	93
89.82	89.2	82.4	90.3	90.3	89.9	88.42	89.4	92.7
89.7	89.1	82.3	90.2	90.2	89.7	88.3	89	92.5
89.62	89	82.2	90.1	90.1	89.6	88.2	88.9	92.2
89.5	88.8	82.1	90	90	89.5	88.08	88.8	91.9
89.4	88.7	81.9	89.9	89.9	89.4	87.96	88.8	91.7
89.22	88.5	81.7	89.8	89.7	89.3	87.8	88.6	91.5
89	88.4	81.5	89.6	89.6	89	87.62	88.3	91.2
88.82	88.3	81.4	89.5	89.4	88.9	87.5	88.2	91.2
88.74	88.2	81.4	89.5	89.4	88.9	87.48	88.2	91
88.66	88.1	81.3	89.5	89.3	88.8	87.4	88	90.8
88.54	88	81.3	89.3	89.2	88.7	87.3	87.8	90.6
88.38	87.8	81.1	89.2	89.1	88.6	87.16	87.5	90.4
88.2	87.7	80.9	89.1	89	88.5	87.04	87.1	90.3
88.06	87.5	80.8	88.9	88.8	88.2	86.84	87	90.2
87.92	87.4	80.7	88.8	88.7	88.1	86.74	86.9	89.8
87.78	87.3	80.6	88.8	88.6	88	86.66	86.6	89.4
87.6	87.1	80.5	88.7	88.5	87.9	86.54	86.2	89.2
87.4	86.9	80.3	88.6	88.4	87.7	86.38	86.1	89
87.2	86.8	80.1	88.4	88.2	87.5	86.2	86	88.8
87	86.6	79.9	88.2	88	87.3	86	85.8	88.4
86.9	86.5	79.8	88.2	87.9	87.2	85.92	85.6	88.1
86.72	86.4	79.8	88.1	87.7	87.1	85.82	85.3	87.8
86.48	86.2	79.5	87.9	87.5	86.9	85.6	84.9	87.4
86.26	86	79.3	87.8	87.4	86.7	85.44	84.7	87.3

86.04	85.8	79.1	87.6	87.2	86.5	85.24	84.5	87.2
85.86	85.6	78.9	87.5	87.1	86.4	85.1	84.3	86.9
85.68	85.4	78.7	87.4	86.9	86.2	84.92	84.2	86.5
85.42	85.2	78.5	87.3	86.7	86.1	84.76	83.8	86
85.18	85	78.4	87.1	86.5	85.8	84.56	83.1	85.5
84.94	84.8	78.3	86.9	86.4	85.6	84.4	82.8	85.2
84.72	84.6	78.1	86.8	86.2	85.4	84.22	82.6	84.9
84.4	84.3	77.8	86.5	85.9	85.2	83.94	81.9	84.2
84.1	84.1	77.5	86.4	85.7	84.9	83.72	81.2	83.5
83.82	83.8	77.3	86.2	85.5	84.6	83.48	80.7	83.2
83.52	83.6	76.9	86	85.3	84.4	83.24	80.3	82.8
83.2	83.3	76.7	85.8	85.1	84.1	83	80	82.3
82.86	83.1	76.5	85.6	84.8	83.8	82.76	79.6	81.8
82.5	82.8	76.3	85.3	84.5	83.6	82.5	78.9	81.1
82.14	82.5	76.1	85.1	84.3	83.3	82.26	78.3	80.6
81.76	82.2	75.8	84.9	84	83	81.98	78	80.3
81.44	81.9	75.6	84.7	83.8	82.7	81.74	77.6	79.7
81.04	81.6	75.4	84.5	83.5	82.4	81.48	77.1	79.2
80.58	81.3	75	84.2	83.1	82	81.12	76.7	78.8
80.12	81	74.7	83.9	82.7	81.6	80.78	76.1	78.4
79.64	80.5	74.4	83.8	82.4	81.3	80.48	75.4	77.8
79.2	80	74.2	83.5	82.1	81	80.16	74.6	76.6
78.8	79.6	73.9	83.3	81.8	80.7	79.86	73.6	75.5
78.3	79.2	73.6	83	81.5	80.3	79.52	72.6	75
77.76	78.8	73.3	82.8	81	79.9	79.16	71.6	74.1
77.2	78.4	73	82.4	80.6	79.4	78.76	70.7	73
76.64	78	72.6	82.1	80.2	79	78.38	69.8	72.3
76.22	77.6	72.3	82	79.9	78.8	78.12	68.9	71.6

B23	B24	B25	B2
96.2	88.8	84.8	91.34
96.7	89	84.9	91.58
96.8	89.2	85	91.7
96.5	89	85	91.56
95.9	88.8	84.8	91.34
95.6	88.7	84.5	91.14
96.1	88.6	84.6	91.24
96.2	88.4	84.6	91.18
95.8	88.3	84.5	91.02
95.7	88.2	84.4	90.94
95.7	88.1	84.4	90.9
95.8	88.2	84.5	90.94
95.7	88.3	84.4	90.9
95.5	88.1	84.3	90.72
95.3	88.1	84.2	90.58
95.2	88	84.1	90.46
95	87.7	83.9	90.3
94.8	87.6	83.9	90.22
94.8	87.6	84	90.16
94.8	87.5	84	90.02
94.6	87.5	83.9	90
94.1	87.4	83.9	89.88
93.9	87.3	83.9	89.74
94.1	87.4	83.9	89.82
94.2	87.4	84	89.94
93.7	87.4	84	89.72
93.4	87.3	83.7	89.48
93.5	87.1	83.5	89.42
93.2	86.9	83.4	89.24
92.8	86.8	83.3	89
92.6	86.7	83.4	88.84
92.4	86.4	83.7	88.72
92.1	86.1	84	88.58
91.9	85.9	84	88.46
91.6	85.8	83.8	88.26
91.3	85.6	83.6	88
90.9	85.6	83.5	87.88
90.8	85.6	83.6	87.84
90.7	85.4	83.4	87.66
90.6	85.3	83.1	87.48
90.2	85	82.8	87.18
89.7	84.7	82.8	86.92
89.5	84.6	83	86.86
89.2	84.5	82.8	86.64
88.9	84.3	82.6	86.36
88.5	84.1	82.5	86.1
88.1	84	82.1	85.86
87.7	83.8	81.9	85.64
87.4	83.5	81.7	85.36
87.2	83.4	81.5	85.16
87	83.2	81.3	84.92
86.3	82.8	81.2	84.52
85.9	82.7	81.2	84.36

85.7	82.8	81.3	84.3
85.5	82.8	81.1	84.12
85.2	82.8	80.8	83.9
84.3	82.5	80.4	83.4
83.5	81.8	79.9	82.76
83.2	81.4	79.9	82.5
82.9	81.3	79.8	82.3
82.2	80.8	79	81.62
81.4	80.1	78.4	80.92
80.6	79.8	78.2	80.5
80.1	79.5	77.9	80.12
79.7	79.1	77.7	79.76
79.1	79	77.4	79.38
78.2	78.7	76.9	78.76
77.6	78.3	76.6	78.28
77.2	78.1	76.4	78
76.5	78	75.8	77.52
75.6	77.3	75.3	76.9
74.8	76.5	75	76.36
74.2	76.2	74.7	75.92
73.4	76.1	74.7	75.48
72.2	75.8	74.2	74.68
70.9	75.1	73.4	73.7
70	74.6	72.8	73
68.9	74.2	72.3	72.22
67.6	73.5	71.8	71.32
66.5	72.8	71.4	70.56
65.5	72.4	71.1	69.9
