# Supplementary material for: Changes in the Biomechanical Properties of Corneal Stromal Lens after Collagen Crosslinking Induced by EDC-NHS
Source: J Ophthalmol. 2024 May 17;2024:9943458. doi: 10.1155/2024/9943458 (PMC11126343; doi:10.1155/2024/9943458)
Supplement: Supplementary Materials — In this study, the preservation methods of corneal stromal lens, collagen crosslinking methods, and inflation testing were based on the previous research results of our team [16] and the research results of Matthew et al. [17]. [file 9943458.f1.zip › Experimental data of lens transmittance detected by wavelength scanning.pdf]

WL(nm)	Con1	Con2	Con3	Con4	Con5	Con	SD	cx111
780	94.7	93.7	96.2	96.6	97	95.64	1.39	98.2
777.5	95.1	94.1	96.4	96.7	97.2	95.9	1.27	98.3
775	95.4	94.2	96.5	96.8	97.4	96.06	1.27	98.5
772.5	95.4	94	96.6	96.8	97.4	96.04	1.35	98.5
770	95.3	93.8	96.5	96.9	97.2	95.94	1.40	98.5
767.5	95.2	93.6	96.5	96.9	97.2	95.88	1.49	98.4
765	95.1	93.5	96.5	96.8	97.2	95.82	1.52	98.3
762.5	95.1	93.6	96.5	96.8	97.2	95.84	1.48	98.4
760	95.2	93.7	96.5	96.9	97.2	95.9	1.45	98.3
757.5	95.1	93.8	96.5	96.9	97.3	95.92	1.45	98.3
755	95.1	93.8	96.5	96.8	97.3	95.9	1.43	98.2
752.5	95.1	93.8	96.4	96.8	97.3	95.88	1.42	98.2
750	95	93.8	96.3	96.8	97.2	95.82	1.40	98.3
747.5	95.1	93.8	96.3	96.7	97.2	95.82	1.37	98.4
745	95.1	93.7	96.3	96.7	97.2	95.8	1.41	98.3
742.5	95	93.6	96.4	96.7	97.2	95.78	1.47	98.3
740	94.9	93.5	96.3	96.6	97.2	95.7	1.49	98.3
737.5	94.8	93.6	96.3	96.6	97.2	95.7	1.47	98.3
735	94.8	93.6	96.4	96.7	97.2	95.74	1.50	98.3
732.5	94.9	93.5	96.4	96.7	97.1	95.72	1.49	98.3
730	94.9	93.5	96.3	96.6	97.2	95.7	1.49	98.2
727.5	94.9	93.5	96.2	96.5	97.2	95.66	1.47	98.3
725	94.9	93.6	96.2	96.6	97.1	95.68	1.42	98.3
722.5	95	93.5	96.2	96.6	97.2	95.7	1.47	98.4
720	94.9	93.5	96.2	96.5	97.2	95.66	1.47	98.4
717.5	94.9	93.5	96.2	96.6	97.1	95.66	1.46	98.3
715	94.8	93.5	96.1	96.5	97	95.58	1.42	98.2
712.5	94.8	93.4	96.1	96.5	97.1	95.58	1.48	98.1
710	94.8	93.4	96.1	96.5	97.1	95.58	1.48	98.2
707.5	94.9	93.4	96.1	96.5	97	95.58	1.44	98.2
705	94.8	93.4	96.2	96.4	97	95.56	1.45	98.1
702.5	94.7	93.4	96.1	96.4	97	95.52	1.45	98.1
700	94.7	93.3	96.1	96.4	97	95.5	1.49	98.1
697.5	94.7	93.3	96.1	96.4	96.9	95.48	1.47	98.1
695	94.7	93.2	96.1	96.3	96.9	95.44	1.49	98
692.5	94.7	93.2	96.2	96.3	96.9	95.46	1.50	98
690	94.6	93.2	96.2	96.3	96.9	95.44	1.51	98.1
687.5	94.6	93.2	96.1	96.3	96.8	95.4	1.48	98.1
685	94.5	93.1	96.1	96.3	96.8	95.36	1.53	98.1
682.5	94.5	93.1	96.1	96.3	96.8	95.36	1.53	98.1
680	94.5	93.2	96	96.3	96.8	95.36	1.48	98
677.5	94.5	93.2	96	96.2	96.8	95.34	1.47	98
675	94.5	93.1	96	96.2	96.8	95.32	1.50	98
672.5	94.5	93.1	96	96.1	96.7	95.28	1.46	97.9
670	94.5	93.1	96	96.1	96.7	95.28	1.46	97.9
667.5	94.4	93	96	96.1	96.7	95.24	1.51	98
665	94.4	92.9	96	96.1	96.7	95.22	1.55	98
662.5	94.3	93	96	96.1	96.7	95.22	1.53	98
660	94.4	93	95.9	96	96.7	95.2	1.49	98
657.5	94.4	92.9	95.9	96	96.7	95.18	1.53	97.9
655	94.3	92.7	95.9	96	96.6	95.1	1.59	97.9
652.5	94.3	92.7	95.9	96	96.6	95.1	1.59	97.9
650	94.3	92.6	95.9	96	96.5	95.06	1.60	97.9

647.5	94.2	92.5	95.9	95.9	96.5	95	1.64	97.9
645	94.3	92.6	95.9	95.9	96.5	95.04	1.59	97.8
642.5	94.3	92.5	95.8	95.9	96.5	95	1.62	97.8
640	94.2	92.5	95.8	95.8	96.5	94.96	1.61	97.8
637.5	94.2	92.5	95.8	95.8	96.5	94.96	1.61	97.8
635	94.2	92.4	95.8	95.8	96.5	94.94	1.65	97.8
632.5	94.1	92.3	95.7	95.7	96.4	94.84	1.65	97.7
630	94.1	92.3	95.7	95.7	96.4	94.84	1.65	97.7
627.5	94.1	92.4	95.7	95.7	96.4	94.86	1.61	97.7
625	94.1	92.3	95.8	95.7	96.4	94.86	1.67	97.6
622.5	93.9	92.2	95.7	95.7	96.4	94.78	1.71	97.6
620	93.8	92.1	95.7	95.6	96.4	94.72	1.75	97.6
617.5	93.8	92	95.8	95.6	96.3	94.7	1.78	97.7
615	93.9	92	95.7	95.5	96.3	94.68	1.74	97.7
612.5	93.9	92.1	95.7	95.5	96.3	94.7	1.70	97.7
610	93.9	92	95.7	95.5	96.3	94.68	1.74	97.6
607.5	93.8	91.9	95.7	95.5	96.2	94.62	1.77	97.6
605	93.8	91.8	95.7	95.4	96.2	94.58	1.79	97.6
602.5	93.7	91.7	95.6	95.3	96.1	94.48	1.79	97.5
600	93.7	91.6	95.6	95.3	96	94.44	1.81	97.4
597.5	93.7	91.7	95.6	95.3	96.1	94.48	1.79	97.5
595	93.7	91.7	95.6	95.3	96.1	94.48	1.79	97.6
592.5	93.7	91.7	95.5	95.4	96.1	94.48	1.79	97.6
590	93.6	91.6	95.5	95.4	96	94.42	1.82	97.5
587.5	93.6	91.4	95.5	95.3	96	94.36	1.88	97.5
585	93.6	91.3	95.5	95.2	96	94.32	1.91	97.5
582.5	93.7	91.4	95.5	95.1	95.9	94.32	1.83	97.4
580	93.6	91.4	95.5	95.2	95.9	94.32	1.85	97.4
577.5	93.4	91.3	95.5	95.1	96	94.26	1.92	97.3
575	93.4	91.2	95.4	95	95.9	94.18	1.91	97.3
572.5	93.4	91.2	95.4	95	95.9	94.18	1.91	97.4
570	93.4	91.2	95.3	95	95.8	94.14	1.87	97.3
567.5	93.4	91.2	95.3	94.9	95.8	94.12	1.86	97.3
565	93.3	91.1	95.2	94.9	95.7	94.04	1.87	97.3
562.5	93.3	90.9	95.2	94.9	95.7	94	1.95	97.3
560	93.2	90.8	95.2	94.8	95.7	93.94	1.99	97.2
557.5	93.1	90.7	95.2	94.7	95.7	93.88	2.03	97.2
555	93.2	90.8	95.1	94.7	95.7	93.9	1.96	97.2
552.5	93.1	90.8	95.1	94.6	95.6	93.84	1.94	97.1
550	93	90.7	95.1	94.6	95.6	93.8	1.99	97.1
547.5	93	90.6	95.1	94.6	95.6	93.78	2.03	97.1
545	93	90.6	95	94.6	95.5	93.74	1.99	97.1
542.5	93	90.6	95	94.5	95.5	93.72	1.98	97.1
540	93	90.6	94.9	94.5	95.4	93.68	1.94	97
537.5	92.9	90.5	94.9	94.4	95.4	93.62	1.98	97
535	92.8	90.4	94.8	94.4	95.4	93.56	2.01	97
532.5	92.8	90.4	94.8	94.3	95.3	93.52	1.98	97
530	92.8	90.3	94.8	94.3	95.2	93.48	2.00	96.9
527.5	92.8	90.3	94.8	94.3	95.2	93.48	2.00	96.9
525	92.7	90.3	94.8	94.3	95.1	93.44	1.98	96.8
522.5	92.6	90.1	94.7	94.2	95.1	93.34	2.05	96.8
520	92.6	90	94.7	94.1	95.1	93.3	2.07	96.8
517.5	92.5	90	94.6	94.1	95.1	93.26	2.07	96.8
515	92.5	89.9	94.6	94	95	93.2	2.07	96.8

512.5	92.6	89.9	94.5	94	95	93.2	2.05	96.8
510	92.7	89.9	94.5	93.9	94.9	93.18	2.01	96.7
507.5	92.6	89.7	94.4	93.9	94.9	93.1	2.08	96.7
505	92.4	89.5	94.4	93.9	94.8	93	2.16	96.7
502.5	92.3	89.4	94.3	93.7	94.7	92.88	2.15	96.6
500	92.3	89.4	94.2	93.7	94.7	92.86	2.13	96.6
497.5	92.2	89.3	94.2	93.6	94.7	92.8	2.17	96.5
495	92.1	89.2	94.2	93.6	94.6	92.74	2.19	96.5
492.5	92.1	89	94.1	93.5	94.5	92.64	2.23	96.5
490	92	89.1	94.1	93.5	94.5	92.64	2.19	96.4
487.5	92.1	89.1	94.1	93.4	94.4	92.62	2.16	96.4
485	92	89	94.1	93.3	94.4	92.56	2.20	96.4
482.5	92	89	94.1	93.3	94.3	92.54	2.18	96.3
480	91.9	89	93.9	93.2	94.2	92.44	2.12	96.2
477.5	92	88.8	93.9	93.1	94.2	92.4	2.19	96.2
475	92	88.7	93.8	93	94.1	92.32	2.18	96.2
472.5	91.9	88.6	93.8	92.9	94	92.24	2.20	96.1
470	91.7	88.4	93.7	92.9	94	92.14	2.27	96.1
467.5	91.6	88.3	93.6	92.9	93.9	92.06	2.28	96
465	91.6	88.3	93.6	92.8	93.8	92.02	2.25	96
462.5	91.6	88.2	93.5	92.7	93.7	91.94	2.25	96
460	91.6	88.2	93.5	92.7	93.7	91.94	2.25	95.9
457.5	91.6	88	93.4	92.6	93.6	91.84	2.29	95.9
455	91.5	87.8	93.3	92.4	93.5	91.7	2.32	95.8
452.5	91.4	87.8	93.3	92.3	93.4	91.64	2.30	95.8
450	91.3	87.7	93.2	92.3	93.4	91.58	2.32	95.7
447.5	91.2	87.5	93.2	92.2	93.3	91.48	2.38	95.7
445	91.2	87.4	93.1	92.1	93.1	91.38	2.36	95.6
442.5	91	87.2	93	92	93	91.24	2.41	95.6
440	90.9	87.1	92.9	92	93	91.18	2.43	95.5
437.5	90.8	87	92.8	91.9	92.9	91.08	2.43	95.4
435	90.8	86.8	92.7	91.7	92.8	90.96	2.46	95.4
432.5	90.8	86.6	92.6	91.5	92.7	90.84	2.50	95.3
430	90.7	86.5	92.4	91.4	92.5	90.7	2.46	95.3
427.5	90.6	86.4	92.3	91.4	92.4	90.62	2.47	95.2
425	90.5	86.3	92.3	91.2	92.3	90.52	2.48	95.1
422.5	90.5	86.2	92.2	91.2	92.2	90.46	2.49	95
420	90.6	86	92.1	91	92	90.34	2.51	94.9
417.5	90.4	85.8	92	90.9	91.9	90.2	2.55	94.8
415	90.1	85.5	91.8	90.7	91.8	89.98	2.61	94.8
412.5	89.9	85.3	91.7	90.6	91.7	89.84	2.65	94.7
410	89.7	84.9	91.6	90.5	91.5	89.64	2.76	94.6
407.5	89.5	84.7	91.5	90.4	91.3	89.48	2.79	94.6
405	89.5	84.6	91.5	90.3	91.2	89.42	2.81	94.5
402.5	89.4	84.5	91.4	90.1	91.1	89.3	2.80	94.4
400	89.2	84.3	91.3	89.9	91	89.14	2.83	94.3
397.5	89	84	91.1	89.7	90.8	88.92	2.88	94.2
395	88.8	83.7	91	89.6	90.6	88.74	2.95	94.1
392.5	88.6	83.4	90.8	89.5	90.4	88.54	3.00	94
390	88.6	83.3	90.7	89.3	90.2	88.42	2.97	93.9
387.5	88.5	83.2	90.6	89.2	90.1	88.32	2.97	93.8
385	88.5	82.9	90.4	88.9	89.9	88.12	3.02	93.7
382.5	88.4	82.6	90.2	88.7	89.7	87.92	3.06	93.6
380	88.3	82.5	90	88.6	89.5	87.78	3.03	93.6

cxl12	cxl13	cxl14	cxl15	5/2.5		cxl21	cxl22	cxl23	cxl24
97.7	96.9	98.2	96.8	97.56	0.61	97.5	97.4	97.8	96.7
98	97.1	98.4	96.9	97.74	0.62	97.7	97.7	98	96.9
98.1	97.2	98.6	97.1	97.9	0.64	97.9	97.8	98.2	96.9
98.1	97.3	98.5	97.1	97.9	0.59	97.9	97.9	98.2	96.8
98.1	97.2	98.5	97	97.86	0.64	97.7	97.9	98.2	96.9
98.1	97.1	98.4	96.9	97.78	0.65	97.7	97.8	98.2	97
98.1	97.1	98.4	97	97.78	0.60	97.8	97.7	98.1	97.1
98.1	97.2	98.4	97.1	97.84	0.57	97.8	97.8	98.1	97
98	97.2	98.4	97	97.78	0.57	97.7	97.8	98.1	96.8
97.9	97.3	98.4	96.9	97.76	0.58	97.7	97.8	98.1	96.9
98	97.3	98.4	97	97.78	0.54	97.7	97.8	98.1	96.9
98	97.3	98.4	97	97.78	0.54	97.7	97.8	98	96.8
98	97.2	98.3	97	97.76	0.55	97.6	97.8	98	96.8
98	97.2	98.3	97	97.78	0.57	97.7	97.8	98.1	96.8
98	97.2	98.4	97	97.78	0.57	97.7	97.8	98.1	96.9
98.1	97	98.4	97	97.76	0.63	97.8	97.8	98.1	96.9
98.1	97	98.5	97.1	97.8	0.63	97.8	97.9	98.1	96.9
98.1	97	98.5	97.1	97.8	0.63	97.7	97.9	98.1	96.9
98	97.1	98.5	97	97.78	0.62	97.7	97.9	98.1	96.9
98	97.1	98.4	97	97.76	0.60	97.6	97.9	98.1	96.9
98	97.1	98.4	97	97.74	0.58	97.6	97.8	98.1	96.9
98	97.1	98.4	97	97.76	0.60	97.6	97.7	98.1	96.9
98.1	97	98.4	97	97.76	0.63	97.7	97.8	98.2	96.9
98.1	97	98.5	97.1	97.82	0.64	97.6	97.9	98.2	96.9
98	96.9	98.5	97.1	97.78	0.66	97.6	97.9	98.2	96.8
98	96.9	98.5	97.1	97.76	0.64	97.6	97.9	98.2	96.8
97.9	96.9	98.4	97.1	97.7	0.60	97.5	97.8	98.1	96.8
97.9	97	98.3	97	97.66	0.55	97.5	97.8	98.1	96.8
97.9	97.1	98.3	96.9	97.68	0.57	97.5	97.8	98.1	96.9
97.9	97	98.4	96.9	97.68	0.62	97.5	97.7	98.1	96.9
97.9	97	98.4	97	97.68	0.58	97.5	97.7	98	96.9
97.9	97	98.4	96.9	97.66	0.60	97.4	97.7	98	96.9
97.9	97	98.3	96.9	97.64	0.58	97.4	97.7	98	96.8
97.9	97	98.2	96.9	97.62	0.56	97.3	97.7	98	96.8
97.8	97	98.2	96.9	97.58	0.53	97.4	97.7	98	96.8
97.8	97	98.3	96.9	97.6	0.55	97.4	97.7	98.1	96.8
97.9	96.9	98.3	96.9	97.62	0.60	97.4	97.7	98.1	96.8
97.9	96.9	98.3	96.9	97.62	0.60	97.4	97.7	98.1	96.8
97.9	96.9	98.3	96.9	97.62	0.60	97.4	97.7	98.1	96.8
97.8	96.9	98.2	96.9	97.58	0.57	97.3	97.6	98	96.8
97.8	96.8	98.2	96.8	97.52	0.60	97.3	97.7	98	96.7
97.8	96.8	98.2	96.8	97.52	0.60	97.3	97.7	98	96.7
97.7	96.8	98.2	96.8	97.5	0.59	97.2	97.6	97.9	96.7
97.7	96.8	98.1	96.7	97.44	0.58	97.2	97.5	97.9	96.7
97.8	96.8	98.2	96.8	97.5	0.59	97.3	97.6	97.9	96.7
97.8	96.9	98.2	96.8	97.54	0.58	97.3	97.6	97.9	96.8
97.8	96.9	98.2	96.8	97.54	0.58	97.3	97.7	97.9	96.8
97.8	96.8	98.2	96.8	97.52	0.60	97.3	97.6	98	96.8
97.7	96.8	98.2	96.8	97.5	0.59	97.2	97.6	98	96.8
97.7	96.8	98.2	96.8	97.48	0.58	97.2	97.6	97.9	96.8
97.7	96.8	98.2	96.8	97.48	0.58	97.3	97.6	97.9	96.7
97.7	96.8	98.2	96.8	97.48	0.58	97.2	97.6	97.9	96.7
97.7	96.8	98.2	96.7	97.46	0.60	97.2	97.6	98	96.8

97.7	96.7	98.1	96.7	97.42	0.60	97.1	97.6	98	96.8
97.7	96.7	98.1	96.7	97.4	0.59	97.2	97.6	98	96.8
97.7	96.7	98.1	96.7	97.4	0.59	97.2	97.6	98	96.8
97.7	96.7	98.1	96.8	97.42	0.56	97.2	97.5	98	96.8
97.7	96.7	98.1	96.8	97.42	0.56	97.2	97.5	98	96.7
97.6	96.7	98.1	96.8	97.4	0.55	97.2	97.5	97.9	96.7
97.6	96.7	98	96.7	97.34	0.54	97.1	97.5	97.9	96.7
97.5	96.7	98	96.6	97.3	0.55	97.1	97.5	97.9	96.7
97.5	96.7	98	96.6	97.3	0.55	97	97.4	97.8	96.7
97.5	96.7	98	96.6	97.28	0.54	97	97.4	97.8	96.7
97.4	96.7	98	96.6	97.26	0.54	96.9	97.4	97.9	96.7
97.4	96.7	98	96.7	97.28	0.51	96.6	97.5	98	96.7
97.4	96.7	97.9	96.7	97.28	0.50	96.5	97.6	98.1	96.7
97.4	96.7	97.9	96.8	97.3	0.48	96.5	97.6	98.1	96.7
97.5	96.6	97.9	96.8	97.3	0.51	96.5	97.6	98.1	96.8
97.4	96.6	97.9	96.7	97.24	0.51	96.5	97.6	98	96.8
97.4	96.6	97.8	96.7	97.22	0.48	96.4	97.5	97.9	96.7
97.4	96.5	97.9	96.7	97.22	0.53	96.4	97.5	98	96.7
97.4	96.5	97.9	96.6	97.18	0.54	96.4	97.5	97.9	96.6
97.3	96.5	97.8	96.6	97.12	0.50	96.3	97.4	97.8	96.6
97.4	96.5	97.8	96.6	97.16	0.52	96.3	97.5	97.9	96.7
97.4	96.5	97.9	96.7	97.22	0.53	96.4	97.5	98	96.7
97.4	96.4	97.9	96.7	97.2	0.56	96.3	97.5	98.1	96.6
97.3	96.4	97.8	96.7	97.14	0.52	96.3	97.5	98	96.6
97.3	96.5	97.8	96.6	97.14	0.51	96.2	97.5	97.9	96.6
97.3	96.5	97.8	96.7	97.16	0.49	96.3	97.5	98	96.6
97.3	96.4	97.8	96.7	97.12	0.50	96.3	97.5	98	96.7
97.2	96.4	97.8	96.7	97.1	0.50	96.2	97.4	97.9	96.7
97.2	96.4	97.8	96.6	97.06	0.50	96.1	97.4	97.8	96.7
97.2	96.4	97.8	96.6	97.06	0.50	96.1	97.3	97.7	96.7
97.3	96.4	97.8	96.6	97.1	0.52	96.1	97.3	97.8	96.6
97.2	96.3	97.7	96.6	97.02	0.50	96.1	97.3	97.8	96.6
97.2	96.3	97.7	96.5	97	0.52	96.1	97.3	97.7	96.6
97.2	96.3	97.7	96.6	97.02	0.50	96.1	97.3	97.7	96.7
97.2	96.4	97.7	96.6	97.04	0.48	96	97.3	97.7	96.6
97.1	96.3	97.6	96.5	96.94	0.48	95.9	97.2	97.7	96.6
97.1	96.3	97.6	96.4	96.92	0.50	95.9	97.1	97.6	96.6
97	96.3	97.6	96.4	96.9	0.49	95.9	97.1	97.6	96.6
97	96.3	97.6	96.4	96.88	0.48	95.9	97.1	97.6	96.6
97	96.2	97.6	96.5	96.88	0.49	95.9	97.1	97.6	96.6
97.1	96.2	97.6	96.5	96.9	0.49	95.9	97.1	97.6	96.7
97	96.2	97.6	96.5	96.88	0.49	95.9	97.1	97.5	96.7
97	96.2	97.5	96.4	96.84	0.48	95.9	97.1	97.5	96.7
96.9	96.2	97.5	96.4	96.8	0.46	95.8	97	97.4	96.7
96.9	96.2	97.5	96.4	96.8	0.46	95.8	97	97.4	96.7
96.9	96.2	97.5	96.4	96.8	0.46	95.8	97	97.4	96.7
96.9	96.1	97.4	96.4	96.76	0.46	95.8	97	97.4	96.7
96.9	96.1	97.4	96.4	96.74	0.45	95.8	97	97.4	96.7
96.9	96.1	97.4	96.3	96.72	0.47	95.7	96.9	97.4	96.7
96.8	96.1	97.3	96.3	96.66	0.42	95.6	96.9	97.3	96.6
96.8	96.1	97.3	96.3	96.66	0.42	95.6	96.9	97.3	96.6
96.8	96.1	97.4	96.3	96.68	0.45	95.6	96.9	97.3	96.6
96.8	96.1	97.3	96.3	96.66	0.42	95.6	96.9	97.3	96.5
96.7	96.1	97.3	96.3	96.64	0.42	95.6	96.9	97.3	96.5

96.7	96.1	97.3	96.3	96.64	0.42	95.6	96.9	97.3	96.6
96.7	96	97.3	96.3	96.6	0.44	95.5	96.8	97.3	96.6
96.7	96	97.3	96.3	96.6	0.44	95.5	96.8	97.2	96.5
96.7	96	97.3	96.3	96.6	0.44	95.5	96.8	97.2	96.5
96.7	96	97.2	96.2	96.54	0.42	95.5	96.7	97.1	96.5
96.6	96	97.2	96.2	96.52	0.41	95.4	96.7	97.1	96.5
96.6	95.9	97.2	96.1	96.46	0.45	95.4	96.6	97.1	96.5
96.6	95.9	97.2	96.1	96.46	0.45	95.4	96.6	97	96.5
96.6	95.9	97.1	96.1	96.44	0.42	95.4	96.6	97	96.5
96.5	95.8	97.1	96.1	96.38	0.44	95.3	96.5	96.9	96.4
96.4	95.8	97	96	96.32	0.41	95.2	96.5	96.8	96.4
96.4	95.8	97	96	96.32	0.41	95.2	96.4	96.8	96.4
96.4	95.8	97	96	96.3	0.41	95.1	96.4	96.8	96.3
96.4	95.7	96.9	96	96.24	0.40	95.1	96.4	96.8	96.3
96.3	95.7	96.9	96	96.22	0.40	95.1	96.4	96.8	96.3
96.3	95.6	96.9	95.9	96.18	0.44	95.1	96.4	96.8	96.3
96.3	95.6	96.9	95.9	96.16	0.44	95	96.3	96.7	96.2
96.2	95.6	96.8	95.9	96.12	0.40	95	96.2	96.7	96.2
96.2	95.6	96.8	95.8	96.08	0.41	94.9	96.2	96.6	96.2
96.2	95.6	96.8	95.8	96.08	0.41	94.9	96.1	96.5	96.2
96.2	95.5	96.7	95.8	96.04	0.40	94.8	96.1	96.5	96.1
96.1	95.5	96.7	95.8	96	0.40	94.8	96	96.5	96.1
96.1	95.4	96.6	95.8	95.96	0.39	94.8	96	96.5	96.1
96	95.4	96.6	95.7	95.9	0.40	94.7	96	96.4	96.1
96	95.4	96.6	95.7	95.9	0.40	94.7	95.9	96.4	96.1
95.9	95.4	96.5	95.7	95.84	0.37	94.6	95.9	96.3	96
95.9	95.3	96.5	95.6	95.8	0.40	94.6	95.8	96.2	96
95.9	95.3	96.4	95.6	95.76	0.37	94.5	95.7	96.2	95.9
95.8	95.3	96.4	95.5	95.72	0.38	94.5	95.6	96.1	95.9
95.8	95.2	96.3	95.5	95.66	0.37	94.4	95.6	96.1	95.8
95.7	95.2	96.3	95.5	95.62	0.38	94.4	95.5	96.1	95.8
95.6	95.2	96.3	95.4	95.58	0.38	94.3	95.5	96	95.8
95.6	95.1	96.2	95.4	95.52	0.38	94.2	95.4	95.9	95.7
95.5	95.1	96.2	95.3	95.48	0.38	94.2	95.3	95.9	95.7
95.5	95	96.1	95.3	95.42	0.38	94.2	95.2	95.8	95.7
95.4	94.9	96	95.2	95.32	0.38	94	95.1	95.7	95.6
95.3	94.8	95.9	95.2	95.24	0.37	93.9	95.1	95.6	95.5
95.2	94.8	95.9	95.1	95.18	0.39	93.8	95	95.6	95.5
95.2	94.7	95.8	95	95.1	0.39	93.8	94.9	95.5	95.4
95.1	94.7	95.7	95	95.06	0.35	93.7	94.8	95.4	95.3
95.1	94.6	95.7	95	95.02	0.39	93.7	94.8	95.4	95.3
95	94.6	95.7	94.9	94.96	0.40	93.7	94.8	95.3	95.3
95	94.6	95.6	94.9	94.94	0.37	93.7	94.7	95.2	95.2
94.9	94.5	95.6	94.8	94.86	0.40	93.6	94.6	95.2	95.2
94.8	94.4	95.5	94.8	94.78	0.40	93.5	94.5	95.1	95.1
94.7	94.4	95.4	94.7	94.7	0.38	93.5	94.5	95	95.1
94.6	94.3	95.3	94.7	94.62	0.39	93.4	94.4	95	95
94.6	94.3	95.2	94.6	94.56	0.37	93.3	94.2	94.8	95
94.5	94.2	95.2	94.5	94.48	0.41	93.2	94.1	94.7	94.8
94.4	94.1	95.1	94.5	94.4	0.41	93.1	94	94.6	94.7
94.2	94	94.9	94.4	94.26	0.38	92.9	93.8	94.5	94.7
94.2	94	94.8	94.3	94.2	0.36	92.9	93.7	94.4	94.6
94.1	93.9	94.8	94.3	94.14	0.40	92.8	93.6	94.4	94.6
94	93.8	94.7	94.2	94.06	0.38	92.8	93.5	94.3	94.6

cxl25	5/5.0		cxl31	cxl32	cxl33	cxl34	cxl35	10/5.0	
97.5	97.38	0.37	96.5	98.2	98.2	98.5	96.6	97.6	0.86
97.7	97.6	0.37	96.7	98.5	98.5	98.8	96.9	97.88	0.89
97.7	97.7	0.43	96.8	98.8	98.6	98.9	97.1	98.04	0.90
97.7	97.7	0.48	96.7	98.8	98.5	98.8	97	97.96	0.92
97.7	97.68	0.43	96.8	98.7	98.5	98.8	97	97.96	0.87
97.8	97.7	0.39	96.8	98.7	98.4	98.8	96.9	97.92	0.88
97.8	97.7	0.33	96.8	98.7	98.4	98.7	96.9	97.9	0.86
97.8	97.7	0.37	96.8	98.7	98.5	98.7	97	97.94	0.85
97.7	97.62	0.44	96.8	98.7	98.5	98.8	97.1	97.98	0.85
97.7	97.64	0.40	96.8	98.7	98.4	98.8	97.1	97.96	0.84
97.8	97.66	0.40	96.7	98.6	98.4	98.7	97.1	97.9	0.83
97.8	97.62	0.42	96.6	98.6	98.4	98.7	97	97.86	0.88
97.7	97.58	0.41	96.7	98.7	98.5	98.7	96.9	97.9	0.90
97.7	97.62	0.44	96.8	98.7	98.4	98.7	96.9	97.9	0.86
97.7	97.64	0.40	96.7	98.6	98.4	98.6	96.9	97.84	0.85
97.7	97.66	0.40	96.7	98.6	98.4	98.7	96.9	97.86	0.87
97.6	97.66	0.41	96.7	98.7	98.4	98.7	96.9	97.88	0.89
97.7	97.66	0.41	96.7	98.6	98.5	98.7	96.9	97.88	0.89
97.7	97.66	0.41	96.8	98.6	98.5	98.6	97	97.9	0.82
97.7	97.64	0.41	96.9	98.7	98.4	98.6	97.1	97.94	0.78
97.6	97.6	0.39	96.8	98.7	98.4	98.6	97	97.9	0.82
97.5	97.56	0.39	96.8	98.6	98.4	98.7	97	97.9	0.82
97.5	97.62	0.43	96.8	98.5	98.4	98.8	97	97.9	0.83
97.6	97.64	0.43	96.9	98.5	98.3	98.7	97	97.88	0.77
97.6	97.62	0.47	96.9	98.5	98.3	98.6	97	97.86	0.75
97.6	97.62	0.47	96.9	98.6	98.3	98.6	97	97.88	0.77
97.5	97.54	0.43	96.8	98.6	98.4	98.6	96.9	97.86	0.83
97.6	97.56	0.43	96.8	98.6	98.4	98.6	96.9	97.86	0.83
97.6	97.58	0.40	96.8	98.6	98.4	98.7	97	97.9	0.82
97.6	97.56	0.39	96.8	98.6	98.4	98.6	97	97.88	0.81
97.6	97.54	0.36	96.9	98.6	98.4	98.7	97	97.92	0.80
97.6	97.52	0.37	96.8	98.6	98.4	98.7	97	97.9	0.82
97.6	97.5	0.40	96.8	98.6	98.4	98.7	97	97.9	0.82
97.5	97.46	0.40	96.8	98.6	98.3	98.6	96.9	97.84	0.82
97.5	97.48	0.40	96.7	98.6	98.3	98.6	96.9	97.82	0.84
97.5	97.5	0.42	96.8	98.6	98.3	98.6	96.9	97.84	0.82
97.6	97.52	0.43	96.8	98.5	98.3	98.6	97	97.84	0.78
97.6	97.52	0.43	96.8	98.5	98.3	98.6	97	97.84	0.78
97.6	97.52	0.43	96.8	98.5	98.3	98.6	96.9	97.82	0.80
97.6	97.46	0.40	96.8	98.5	98.3	98.6	96.9	97.82	0.80
97.5	97.44	0.44	96.8	98.5	98.3	98.6	96.9	97.82	0.80
97.5	97.44	0.44	96.7	98.5	98.3	98.6	96.9	97.8	0.82
97.5	97.38	0.41	96.8	98.5	98.3	98.6	96.8	97.8	0.82
97.5	97.36	0.40	96.8	98.4	98.3	98.6	96.9	97.8	0.78
97.5	97.4	0.40	96.8	98.4	98.3	98.6	96.9	97.8	0.78
97.5	97.42	0.37	96.8	98.5	98.3	98.6	96.9	97.82	0.80
97.5	97.44	0.38	96.8	98.4	98.3	98.6	96.9	97.8	0.78
97.5	97.44	0.39	96.9	98.4	98.3	98.6	96.9	97.82	0.76
97.5	97.42	0.40	96.9	98.5	98.3	98.6	96.9	97.84	0.77
97.5	97.4	0.37	96.8	98.5	98.3	98.6	96.9	97.82	0.80
97.5	97.4	0.40	96.8	98.5	98.4	98.5	97	97.84	0.77
97.5	97.38	0.41	96.8	98.5	98.4	98.6	96.9	97.84	0.81
97.5	97.42	0.40	96.8	98.5	98.3	98.6	96.8	97.8	0.82

97.5	97.4	0.41	96.8	98.4	98.3	98.6	96.9	97.8	0.78
97.5	97.42	0.40	96.7	98.4	98.3	98.6	96.9	97.78	0.81
97.4	97.4	0.40	96.7	98.5	98.3	98.6	96.9	97.8	0.82
97.5	97.4	0.39	96.7	98.5	98.3	98.6	96.9	97.8	0.82
97.5	97.38	0.43	96.7	98.4	98.3	98.5	96.9	97.76	0.79
97.4	97.34	0.39	96.7	98.4	98.2	98.5	96.8	97.72	0.80
97.4	97.32	0.40	96.7	98.4	98.2	98.5	96.8	97.72	0.80
97.4	97.32	0.40	96.7	98.4	98.3	98.5	96.8	97.74	0.81
97.4	97.26	0.38	96.7	98.4	98.3	98.5	96.9	97.76	0.79
97.4	97.26	0.38	96.7	98.4	98.2	98.5	96.9	97.74	0.78
97.3	97.24	0.42	96.8	98.4	98.2	98.5	96.9	97.76	0.75
97.4	97.24	0.52	96.9	98.3	98.2	98.6	96.9	97.78	0.73
97.4	97.26	0.59	96.9	98.3	98.2	98.6	96.9	97.78	0.73
97.3	97.24	0.59	96.9	98.3	98.1	98.6	96.8	97.74	0.74
97.3	97.26	0.57	96.9	98.3	98.1	98.6	96.8	97.74	0.74
97.4	97.26	0.54	96.9	98.4	98.2	98.6	96.8	97.78	0.77
97.3	97.16	0.54	96.9	98.4	98.1	98.6	96.8	97.76	0.76
97.3	97.18	0.57	96.8	98.4	98.2	98.6	96.8	97.76	0.79
97.2	97.12	0.56	96.8	98.3	98.1	98.6	96.8	97.72	0.77
97.2	97.06	0.54	96.8	98.3	98	98.5	96.8	97.68	0.74
97.3	97.14	0.57	96.8	98.3	98.1	98.6	96.9	97.74	0.74
97.3	97.18	0.57	96.8	98.4	98.2	98.6	96.8	97.76	0.79
97.2	97.14	0.64	96.8	98.4	98.2	98.7	96.8	97.78	0.82
97.2	97.12	0.61	96.8	98.4	98.1	98.6	96.8	97.74	0.78
97.2	97.08	0.61	96.7	98.3	98	98.5	96.8	97.66	0.76
97.2	97.12	0.61	96.8	98.3	98	98.5	96.8	97.68	0.74
97.2	97.14	0.60	96.8	98.3	98	98.5	96.8	97.68	0.74
97.2	97.08	0.58	96.8	98.3	98	98.5	96.8	97.68	0.74
97.2	97.04	0.59	96.8	98.3	98.1	98.5	96.7	97.68	0.77
97.2	97	0.55	96.8	98.3	98.1	98.6	96.7	97.7	0.79
97.2	97	0.59	96.7	98.3	98.1	98.6	96.7	97.68	0.82
97.2	97	0.59	96.7	98.3	98.1	98.6	96.7	97.68	0.82
97.2	96.98	0.56	96.7	98.2	98.1	98.6	96.7	97.66	0.80
97.2	97	0.55	96.6	98.3	98.1	98.6	96.7	97.66	0.84
97.1	96.94	0.59	96.6	98.2	98	98.5	96.7	97.6	0.79
97.1	96.9	0.61	96.7	98.2	98	98.5	96.7	97.62	0.77
97.1	96.86	0.57	96.7	98.2	98	98.5	96.7	97.62	0.77
97.1	96.86	0.57	96.7	98.2	98	98.6	96.7	97.64	0.79
97.1	96.86	0.57	96.6	98.2	98	98.5	96.7	97.6	0.79
97.1	96.86	0.57	96.6	98.2	98	98.5	96.6	97.58	0.82
97.1	96.88	0.57	96.6	98.2	98.1	98.5	96.6	97.6	0.83
97.1	96.86	0.54	96.6	98.2	98.1	98.5	96.6	97.6	0.83
97.1	96.86	0.54	96.6	98.1	98.1	98.5	96.6	97.58	0.81
97.1	96.8	0.55	96.6	98.1	98.1	98.5	96.6	97.58	0.81
97.1	96.8	0.55	96.5	98.1	98.1	98.5	96.6	97.56	0.84
97.1	96.8	0.55	96.5	98.1	98	98.5	96.6	97.54	0.83
97	96.78	0.54	96.5	98.1	98	98.4	96.6	97.52	0.80
97	96.78	0.54	96.5	98.1	98	98.4	96.6	97.52	0.80
97	96.74	0.57	96.4	98.1	98	98.5	96.6	97.52	0.85
96.9	96.66	0.57	96.4	98.1	98	98.5	96.5	97.5	0.87
96.9	96.66	0.57	96.4	98.1	97.9	98.4	96.5	97.46	0.84
96.9	96.66	0.57	96.4	98	97.9	98.4	96.5	97.44	0.83
96.8	96.62	0.57	96.4	98	97.9	98.4	96.5	97.44	0.83
96.8	96.62	0.57	96.4	98	97.9	98.4	96.4	97.42	0.85

96.8	96.64	0.57	96.4	98	97.9	98.4	96.5	97.44	0.83
96.8	96.6	0.60	96.4	98	97.9	98.4	96.5	97.44	0.83
96.8	96.56	0.57	96.3	98	97.8	98.3	96.5	97.38	0.82
96.8	96.56	0.57	96.3	98	97.8	98.3	96.4	97.36	0.84
96.8	96.52	0.55	96.3	98	97.8	98.3	96.4	97.36	0.84
96.8	96.5	0.58	96.3	97.9	97.8	98.3	96.4	97.34	0.83
96.8	96.48	0.58	96.2	97.9	97.9	98.3	96.3	97.32	0.89
96.7	96.44	0.55	96.2	97.9	97.9	98.3	96.3	97.32	0.89
96.7	96.44	0.55	96.2	97.9	97.9	98.3	96.3	97.32	0.89
96.7	96.36	0.56	96.2	97.9	97.8	98.3	96.3	97.3	0.87
96.7	96.32	0.58	96.1	97.8	97.8	98.3	96.3	97.26	0.89
96.6	96.28	0.56	96.1	97.8	97.8	98.2	96.3	97.24	0.86
96.6	96.24	0.60	96.1	97.8	97.8	98.3	96.3	97.26	0.89
96.5	96.22	0.58	96	97.8	97.7	98.3	96.2	97.2	0.92
96.5	96.22	0.58	96	97.8	97.7	98.2	96.2	97.18	0.90
96.5	96.22	0.58	96	97.8	97.7	98.2	96.2	97.18	0.90
96.4	96.12	0.58	96	97.7	97.7	98.2	96.1	97.14	0.91
96.4	96.1	0.58	95.9	97.7	97.7	98.2	96.1	97.12	0.93
96.4	96.06	0.60	95.9	97.7	97.6	98.2	96.1	97.1	0.92
96.3	96	0.57	95.9	97.7	97.6	98.1	96.1	97.08	0.90
96.3	95.96	0.60	95.9	97.6	97.6	98.1	96	97.04	0.91
96.3	95.94	0.60	95.8	97.6	97.6	98.1	96	97.02	0.93
96.2	95.92	0.58	95.8	97.6	97.6	98.1	96	97.02	0.93
96.2	95.88	0.60	95.8	97.6	97.6	98.1	96	97.02	0.93
96.2	95.86	0.60	95.7	97.6	97.5	98.1	95.9	96.96	0.97
96.2	95.8	0.62	95.7	97.5	97.5	98	95.9	96.92	0.93
96.1	95.74	0.59	95.7	97.5	97.5	98	95.9	96.92	0.93
96	95.66	0.60	95.7	97.5	97.5	98	95.8	96.9	0.96
96	95.62	0.58	95.6	97.5	97.4	98	95.8	96.86	0.97
95.9	95.56	0.60	95.6	97.4	97.4	98	95.8	96.84	0.96
95.9	95.54	0.60	95.5	97.4	97.4	98	95.7	96.8	1.01
95.8	95.48	0.61	95.5	97.4	97.4	97.9	95.7	96.78	0.98
95.8	95.4	0.62	95.4	97.4	97.3	97.9	95.7	96.74	1.00
95.7	95.36	0.61	95.4	97.3	97.3	97.8	95.6	96.68	0.98
95.7	95.32	0.60	95.3	97.3	97.3	97.8	95.5	96.64	1.03
95.6	95.2	0.64	95.3	97.2	97.2	97.7	95.5	96.58	0.98
95.5	95.12	0.63	95.2	97.2	97.1	97.7	95.5	96.54	1.00
95.4	95.06	0.66	95.2	97.1	97.1	97.7	95.4	96.5	1.01
95.4	95	0.64	95.1	97.1	97.1	97.6	95.3	96.44	1.03
95.3	94.9	0.64	95.1	97.1	97	97.6	95.3	96.42	1.02
95.2	94.88	0.62	95	97.1	97	97.6	95.2	96.38	1.07
95.1	94.84	0.60	95	97	97	97.6	95.2	96.36	1.05
95	94.76	0.56	95	97	97	97.6	95.2	96.36	1.05
94.9	94.7	0.59	95	96.9	96.9	97.5	95.1	96.28	1.03
94.9	94.62	0.60	94.9	96.9	96.9	97.5	95.1	96.26	1.05
94.8	94.58	0.58	94.8	96.9	96.8	97.4	95.1	96.2	1.04
94.7	94.5	0.59	94.7	96.8	96.8	97.4	95	96.14	1.08
94.7	94.4	0.61	94.7	96.8	96.8	97.4	94.9	96.12	1.10
94.6	94.28	0.59	94.6	96.7	96.7	97.3	94.9	96.04	1.08
94.4	94.16	0.58	94.6	96.7	96.6	97.3	94.9	96.02	1.07
94.3	94.04	0.64	94.5	96.7	96.6	97.2	94.8	95.96	1.09
94.2	93.96	0.61	94.4	96.6	96.5	97.1	94.7	95.86	1.09
94.1	93.9	0.64	94.3	96.5	96.4	97	94.6	95.76	1.09
94.1	93.86	0.64	94.2	96.5	96.4	97	94.6	95.74	1.12

B21	B22	B23	B24	B25	B2	
97.4	98.6	98.5	97.9	98.8	98.24	0.52
97.7	98.8	98.6	98	99	98.42	0.49
97.8	98.8	98.6	97.9	99.1	98.44	0.51
97.7	98.8	98.6	97.9	98.9	98.38	0.49
97.7	98.9	98.6	97.9	98.8	98.38	0.49
97.6	98.8	98.7	98	98.9	98.4	0.51
97.6	98.8	98.7	97.9	98.8	98.36	0.51
97.6	98.9	98.6	97.9	98.7	98.34	0.50
97.6	98.9	98.6	97.9	98.8	98.36	0.52
97.6	98.9	98.7	97.9	98.8	98.38	0.53
97.5	98.9	98.7	97.8	98.9	98.36	0.59
97.6	98.8	98.7	97.9	98.9	98.38	0.53
97.5	98.8	98.6	97.9	98.8	98.32	0.53
97.5	98.8	98.6	97.8	98.8	98.3	0.54
97.6	98.7	98.6	97.9	98.8	98.32	0.48
97.6	98.7	98.6	98	98.8	98.34	0.46
97.6	98.8	98.7	98	98.8	98.38	0.49
97.7	98.8	98.8	97.9	98.9	98.42	0.51
97.7	98.8	98.7	97.9	98.8	98.38	0.48
97.6	98.8	98.7	97.9	98.8	98.36	0.51
97.6	98.8	98.7	97.9	98.8	98.36	0.51
97.6	98.8	98.7	97.9	98.8	98.36	0.51
97.6	98.8	98.6	97.9	98.7	98.32	0.48
97.6	98.8	98.5	97.7	98.7	98.26	0.51
97.6	98.8	98.5	97.7	98.6	98.24	0.49
97.6	98.8	98.5	97.8	98.6	98.26	0.47
97.5	98.7	98.5	97.7	98.5	98.18	0.48
97.5	98.6	98.5	97.7	98.6	98.18	0.48
97.5	98.7	98.5	97.8	98.6	98.22	0.48
97.6	98.7	98.5	97.8	98.6	98.24	0.45
97.5	98.7	98.5	97.8	98.6	98.22	0.48
97.5	98.7	98.5	97.7	98.5	98.18	0.48
97.5	98.7	98.4	97.7	98.5	98.16	0.47
97.5	98.7	98.4	97.7	98.5	98.16	0.47
97.4	98.6	98.4	97.7	98.5	98.12	0.48
97.5	98.6	98.4	97.7	98.5	98.14	0.45
97.6	98.6	98.4	97.7	98.4	98.14	0.41
97.5	98.6	98.4	97.7	98.4	98.12	0.44
97.4	98.6	98.4	97.7	98.5	98.12	0.48
97.4	98.6	98.3	97.7	98.4	98.08	0.45
97.4	98.6	98.3	97.6	98.4	98.06	0.47
97.4	98.5	98.4	97.7	98.5	98.1	0.46
97.3	98.5	98.4	97.7	98.5	98.08	0.49
97.3	98.5	98.3	97.6	98.4	98.02	0.48
97.4	98.5	98.3	97.6	98.4	98.04	0.45
97.4	98.6	98.3	97.6	98.4	98.06	0.47
97.4	98.6	98.4	97.6	98.4	98.08	0.48
97.4	98.6	98.4	97.6	98.4	98.08	0.48
97.4	98.5	98.4	97.6	98.4	98.06	0.46
97.3	98.5	98.3	97.6	98.3	98	0.46
97.3	98.5	98.3	97.5	98.3	97.98	0.48
97.3	98.4	98.2	97.5	98.3	97.94	0.45
97.3	98.3	98.3	97.6	98.3	97.96	0.43

97.3	98.4	98.3	97.6	98.3	97.98	0.44
97.3	98.4	98.3	97.6	98.2	97.96	0.43
97.3	98.4	98.3	97.5	98.2	97.94	0.45
97.3	98.4	98.2	97.5	98.2	97.92	0.44
97.2	98.3	98.2	97.5	98.2	97.88	0.44
97.2	98.2	98.2	97.5	98.1	97.84	0.41
97.2	98.2	98.2	97.5	98.1	97.84	0.41
97.2	98.2	98.2	97.5	98.1	97.84	0.41
97.2	98.2	98.2	97.5	98.1	97.84	0.41
97.2	98.2	98.2	97.5	98.1	97.84	0.41
97.1	98.2	98.1	97.4	98	97.76	0.43
97.1	98.1	98.1	97.4	97.9	97.72	0.40
97.1	98.1	98.1	97.4	97.9	97.72	0.40
97.1	98.1	98.1	97.4	97.9	97.72	0.40
97.1	98.1	98.1	97.4	97.9	97.72	0.40
97.1	98.1	98	97.3	97.9	97.68	0.40
97.1	98	98	97.3	97.8	97.64	0.37
97	97.9	97.9	97.3	97.8	97.58	0.37
96.9	97.9	97.9	97.2	97.7	97.52	0.40
96.9	97.9	97.8	97.2	97.6	97.48	0.38
96.9	97.9	97.8	97.2	97.7	97.5	0.38
96.9	97.9	97.9	97.2	97.7	97.52	0.40
96.9	97.9	98	97.3	97.7	97.56	0.41
96.9	97.8	97.9	97.2	97.7	97.5	0.38
96.9	97.8	97.8	97.2	97.7	97.48	0.37
96.9	97.8	97.8	97.2	97.6	97.46	0.36
96.8	97.8	97.8	97.2	97.6	97.44	0.39
96.8	97.7	97.8	97.1	97.5	97.38	0.38
96.7	97.7	97.8	97.1	97.5	97.36	0.41
96.7	97.6	97.7	97.1	97.4	97.3	0.36
96.6	97.5	97.7	97	97.4	97.24	0.39
96.7	97.5	97.7	97	97.4	97.26	0.36
96.7	97.5	97.6	97	97.4	97.24	0.34
96.6	97.5	97.6	97	97.4	97.22	0.37
96.6	97.5	97.6	97	97.3	97.2	0.36
96.6	97.4	97.6	96.9	97.3	97.16	0.36
96.5	97.4	97.5	96.9	97.3	97.12	0.37
96.5	97.3	97.5	96.9	97.2	97.08	0.35
96.4	97.2	97.5	96.9	97.2	97.04	0.37
96.4	97.2	97.5	96.9	97.2	97.04	0.37
96.3	97.1	97.5	96.8	97.2	96.98	0.41
96.3	97.1	97.4	96.7	97.1	96.92	0.38
96.2	97	97.4	96.7	97.2	96.9	0.42
96.2	97	97.3	96.7	97.1	96.86	0.38
96.2	97	97.3	96.7	97.1	96.86	0.38
96.2	97	97.3	96.6	97	96.82	0.38
96.1	96.9	97.2	96.6	97	96.76	0.38
96.1	96.8	97.2	96.5	96.9	96.7	0.37
96	96.7	97.1	96.5	96.8	96.62	0.37
95.9	96.7	97.1	96.5	96.8	96.6	0.40
95.9	96.7	97.1	96.4	96.7	96.56	0.40
95.8	96.6	97	96.4	96.7	96.5	0.40
95.7	96.5	97	96.4	96.7	96.46	0.43
95.7	96.5	96.9	96.4	96.6	96.42	0.40

95.6	96.4	96.8	96.3	96.5	96.32	0.40
95.5	96.4	96.7	96.2	96.4	96.24	0.40
95.5	96.3	96.7	96.2	96.3	96.2	0.39
95.4	96.1	96.6	96.1	96.2	96.08	0.39
95.2	96	96.5	96	96.1	95.96	0.42
95.1	95.9	96.4	96	96.1	95.9	0.43
94.9	95.7	96.3	95.9	95.9	95.74	0.46
94.7	95.5	96.1	95.8	95.8	95.58	0.48
94.6	95.3	96	95.7	95.7	95.46	0.48
94.4	95.1	95.8	95.5	95.5	95.26	0.48
94.1	94.9	95.6	95.4	95.3	95.06	0.53
93.9	94.8	95.5	95.3	95.1	94.92	0.56
93.7	94.6	95.3	95.2	94.9	94.74	0.57
93.5	94.4	95.1	95.1	94.7	94.56	0.59
93.3	94.2	95	95	94.6	94.42	0.63
93.1	94.1	94.8	94.9	94.5	94.28	0.65
92.9	93.9	94.7	94.8	94.3	94.12	0.69
92.8	93.8	94.6	94.7	94.2	94.02	0.69
92.6	93.6	94.4	94.6	94.1	93.86	0.71
92.5	93.5	94.3	94.5	94	93.76	0.71
92.4	93.4	94.2	94.4	93.9	93.66	0.71
92.2	93.3	94.1	94.3	93.8	93.54	0.75
92.1	93.1	94	94.2	93.7	93.42	0.76
92	93	93.9	94.2	93.6	93.34	0.78
91.9	92.9	93.7	94.1	93.5	93.22	0.77
91.7	92.8	93.6	94	93.4	93.1	0.80
91.6	92.6	93.5	93.9	93.3	92.98	0.81
91.5	92.5	93.5	93.8	93.2	92.9	0.82
91.4	92.5	93.4	93.7	93.2	92.84	0.82
91.3	92.4	93.2	93.7	93	92.72	0.82
91.2	92.2	93.2	93.6	93	92.64	0.85
91.1	92.1	93.1	93.5	92.9	92.54	0.85
91	92	92.9	93.4	92.8	92.42	0.84
90.8	91.9	92.8	93.3	92.7	92.3	0.87
90.6	91.8	92.6	93.2	92.6	92.16	0.90
90.4	91.5	92.3	93	92.4	91.92	0.90
90.1	91.4	92.1	92.9	92.2	91.74	0.95
89.9	91.1	91.8	92.7	91.9	91.48	0.94
89.6	90.9	91.6	92.6	91.7	91.28	1.00
89.3	90.7	91.3	92.4	91.5	91.04	1.03
89.1	90.5	91	92.2	91.4	90.84	1.03
88.8	90.2	90.7	92	91.3	90.6	1.08
88.6	90	90.4	91.9	91.1	90.4	1.11
88.3	89.7	90.2	91.7	90.9	90.16	1.15
88	89.4	89.9	91.5	90.6	89.88	1.18
87.7	89.2	89.6	91.3	90.3	89.62	1.20
87.5	88.9	89.4	91.1	90.1	89.4	1.20
87.2	88.6	89.1	90.9	89.9	89.14	1.24
86.9	88.3	88.8	90.6	89.7	88.86	1.26
86.5	87.9	88.4	90.4	89.3	88.5	1.31
86.1	87.5	88.1	90.1	89	88.16	1.35
85.8	87.1	87.8	89.8	88.6	87.82	1.35
85.5	86.8	87.6	89.6	88.3	87.56	1.38
85.3	86.5	87.5	89.4	88.1	87.36	1.39
