# Supplementary material for: Changes in the Biomechanical Properties of Corneal Stromal Lens after Collagen Crosslinking Induced by EDC-NHS
Source: J Ophthalmol. 2024 May 17;2024:9943458. doi: 10.1155/2024/9943458 (PMC11126343; doi:10.1155/2024/9943458)
Supplement: Supplementary Materials — In this study, the preservation methods of corneal stromal lens, collagen crosslinking methods, and inflation testing were based on the previous research results of our team [16] and the research results of Matthew et al. [17]. [file 9943458.f1.zip › Inflation testing.pdf]

	屈光度（D）	组别	厚度	0	3	6	9
um	-4	Control	15/84		213.866	259.748	275.024
	-5	Control	15/104	0	15.276	61.105	76.381
	-5.125	Control	15/99		320.801	351.354	366.63
	-5.625	Control	15/107	0	61.105	106.934	137.486
	-5.75	Control	15/104	0	213.868	244.42	259.696
	-6.375	Control	15/119	0	290.249	336.078	366.63
	-6.625	Control	15/124	0	214.333	260.79	290.64
	-7		15/128		229.144	259.696	274.973
	-7.25		15/145		290.249	305.525	320.801
	-7.375		15/133		290.249	305.525	336.078
平均	-6.0125	1.1031		0.0000	213.9140	249.1175	270.4339
	-4.125	5/2.5	15/83		15.276	15.276	30.553
	-4.5	5/2.5	15/85	0	0	15.276	15.276
	-5.125	5/2.5	15/100	0	15.276	15.276	30.553
	-5.625	5/2.5	15/106	0	15.276	30.553	45.829
	-6	5/2.5	15/108		15.276	30.553	45.829
	-6.125	5/2.5	15/114	0	0	15.276	30.553
	-6.5	5/2.5	15/119	0	0	15.276	15.276
	-6.75	5/2.5	15/127	0	30.553	76.381	106.934
	-7.375	5/2.5	15/134	0	0	0	15.276
	-7.5	5/2.5	15/142	0	15.276	15.276	30.552
		标准差					
平均	-5.9625	1.1366		0.0000	9.5475	22.9143	36.6631
	-4.375	5/5	15/87		30.553	61.105	76.381
	-4.5	5/5	15/95	0	30.553	76.381	106.934
	-4.875	5/5	15/98	0	152.763	229.144	290.249
	-5.75	5/5	15/103	0	45.829	91.657	122.21
	-5.75	5/5	10/105		320.801	351.354	397.183
	-6.25	5/5	15/109	0	30.553	45.829	76.381
	-6.75	5/5	15/132	0	76.381	122.21	152.7663
	-6.875	5/5	15/133	0	15.276	45.829	76.381
	-7.625	5/5	15/144	0	30.553	61.105	91.657
	-7.625	5/5	15/141	0	91.657	152.763	183.315
平均	-6.0375	1.1990		0.0000	98.2047	127.9386	162.3107
	-4.25	10/5	15/88	0	0	15.276	15.276
	-4.875	10/5	15/103	0	15.276	15.276	15.276
	-4.875	10/5	15/102		0	0	0
	-5.125	10/5	15/110	0	15.276	15.276	15.276
	-6.125	10/5	15/116		15.276	15.276	30.553
	-6.25	10/5	15/123	0	15.276	15.276	30.553
	-6.5	10/5	15/120	0	15.970	30.52	29.81
	-7	10/5	15/133	0	15.276	30.553	30.553
	-7.25	10/5	15/138	0	0	0	0
	-7.625	10/5	15/141	0	15.276	15.276	30.553

平均	-5.9875	1.1494		0.0000	9.5475	15.2729	19.7850
	-4.25	B2	15/88		213.868	366.63	397.183
	-5	B2	15/100		91.657	137.486	168.039
	-5.25	B2	15/108		259.696	290.249	381.906
	-5.625	B2	15/110		229.144	274.973	305.525
	-5.5	B2	15/105		152.763	183.315	198.591
	-6.25	B2	15/125		106.934	168.039	183.315
	-6.5	B2	15/114		76.381	106.934	137.486
	-6.625	B2	15/122		76.381	106.934	106.934
	-7.375	B2	15/134		122.21	168.039	198.591
	-7.75	B2	15/130		198.591	274.973	320.801
	-6.0125	1.0888		0	152.7625	207.7572	239.8371
	-4.25	B2	15/81		320.801	351.354	381.906
	-5		15/99		213.868	259.696	274.973
	-5.25		15/103		213.868	244.42	274.973
	-5.625		15/113		152.763	198.591	229.144
	-5.625		15/106		168.039	213.868	259.696
	-6.25		15/134		61.105	91.657	122.210
	-6.25		15/112		91.657	122.21	152.763
	-7		15/127		137.486	152.763	183.315
	-7.25		15/126		152.763	183.315	198.591
	-7.5		15/138		152.763	183.315	213.868
	-6	1.04583		0	166.5113	200.1189	229.1439

12	15	18	21	24	27	30
305.63	320.906	336.022	336.022	336.022	351.245	351.245
91.657	91.657	122.21	137.486	152.763	168.039	168.039
381.906	381.906	397.183	412.459	412.459	427.735	427.735
168.039	183.315	213.868	229.144	229.144	244.420	259.696
290.249	305.525	305.525	320.801	336.078	336.078	351.354
381.906	397.183	412.459	427.735	443.011	443.011	443.011
307.921	322.509	338.444	337.098	353.032	353.032	367.62
290.249	305.525	320.801	336.078	320.801	320.801	320.801
336.078	336.078	351.354	351.354	351.354	351.354	366.630
351.354	351.354	351.354	366.630	366.630	381.906	381.906
290.4989	299.5958	314.9220	325.4807	330.1294	337.7621	343.8037
30.553	30.553	45.829	45.829	45.829	61.105	61.105
30.553	45.829	61.105	76.381	91.657	106.934	122.21
30.553	45.829	61.105	76.381	76.381	91.657	106.934
61.105	91.657	91.657	106.934	122.210	137.486	152.763
61.105	76.381	91.657	106.934	106.934	122.21	137.486
30.553	30.553	45.829	45.829	61.105	61.105	76.381
30.553	30.553	45.829	45.829	61.105	61.105	76.381
137.486	152.763	183.315	213.868	213.868	229.144	244.42
15.276	15.276	15.276	30.553	45.829	45.829	45.829
30.469	30.386	45.994	45.994	61.187	61.104	61.104
45.8206	54.9780	68.7596	79.4532	88.6105	97.7679	108.4613
91.657	106.934	122.210	122.210	137.486	137.486	137.486
137.486	168.039	198.591	198.591	213.868	229.144	244.42
320.801	336.078	351.354	366.63	381.906	381.906	397.183
152.763	168.039	183.315	198.591	213.868	229.144	229.144
412.459	427.735	427.735	443.011	443.011	458.288	458.288
91.657	106.934	122.21	137.486	152.763	168.039	168.039
168.039	183.315	198.591	213.868	229.144	229.144	244.42
106.934	137.486	152.763	183.315	198.591	198.591	213.868
106.934	137.486	152.763	168.039	183.315	183.315	198.591
198.591	213.868	229.144	244.42	244.42	259.696	259.696
185.2245	204.3200	219.5961	232.9628	246.3296	253.9678	261.6060
30.553	45.829	45.829	61.105	76.381	91.657	91.657
30.553	30.553	45.829	61.105	61.105	76.381	76.381
0	15.276	15.276	15.276	30.553	30.553	30.553
30.553	30.553	30.553	30.553	30.553	45.829	45.829
30.553	45.829	61.105	76.381	91.657	91.657	106.934
45.829	61.105	76.381	76.381	91.657	106.934	106.934
45.07	60.329	59.619	58.91	75.589	90.139	105.399
45.829	45.829	45.829	61.105	76.381	76.381	91.657
0	15.276	15.276	15.276	30.553	30.553	30.553
30.553	45.829	45.829	61.105	61.105	61.105	76.381

28.9493	39.6408	44.1526	51.7197	62.5534	70.1189	76.2278
412.459	427.735	443.011	443.011	443.011	458.288	458.288
183.315	198.591	213.868	229.144	244.420	244.42	244.42
412.459	412.459	412.459	427.735	427.735	443.11	443.011
320.801	336.078	351.354	366.630	366.630	381.906	397.183
213.868	229.144	244.42	244.420	259.696	259.696	274.973
213.868	244.420	244.42	259.696	274.973	290.249	290.249
152.763	168.039	168.39	183.315	183.315	198.591	198.591
122.210	137.486	152.763	152.763	152.763	152.763	183.315
213.868	229.144	244.42	259.696	259.696	274.973	274.973
351.354	366.630	366.63	381.096	381.096	397.183	397.183
259.6965	274.9726	284.1735	294.7506	299.3335	310.1179	316.2186
397.183	412.459	427.735	443.011	443.011	443.011	458.288
290.249	305.525	320.801	320.801	336.078	336.078	351.354
274.973	290.249	290.249	305.525	305.525	320.801	336.078
259.696	274.973	290.249	305.525	320.801	320.801	336.078
274.973	290.249	320.801	320.801	336.078	336.078	351.354
152.763	168.039	183.315	198.591	213.868	244.420	259.696
168.039	183.315	198.591	198.591	213.868	229.144	229.144
183.315	198.591	198.591	213.868	213.868	229.144	229.144
213.868	229.144	244.420	259.696	274.973	274.973	290.249
244.420	244.420	274.973	274.973	290.249	305.525	305.525
245.9479	259.6964	274.9725	284.1382	294.8319	303.9975	314.691

33	36	39	42	45	48
366.575	366.575	382.17	382.223	382.223	397.34
168.039	198.591	198.591	213.868	229.144	244.42
443.011	458.288	443.011	458.288	473.564	488.84
259.696	276.973	274.973	274.973	290.249	290.249
351.354	351.354	351.354	351.354	351.354	366.63
458.288	458.288	473.564	473.564	488.84	488.84
368.294	367.62	397.47	414.078	429.339	430.013
336.078	320.801	320.801	320.801	336.078	336.078
366.630	366.630	366.630	381.906	381.906	381.906
397.183	412.459	412.459	412.459	427.735	427.735
351.5148	357.7579	362.1023	368.3514	379.0432	385.2051
61.105	76.381	76.381	76.381	76.381	91.657
137.486	137.486	152.763	168.039	183.315	198.591
106.934	137.486	137.486	152.763	152.763	168.039
168.039	168.039	183.315	198.591	213.868	213.868
137.486	152.763	168.039	168.039	183.315	183.315
76.381	91.657	91.657	106.934	106.934	122.21
91.657	106.934	106.934	122.21	122.21	122.21
244.42	259.696	259.696	259.696	259.696	259.696
45.829	61.105	61.105	61.105	61.105	76.381
76.297	76.297	91.49	91.49	106.849	106.849
114.5634	126.7844	132.8866	140.5248	146.6436	154.2816
152.763	152.763	152.763	168.039	168.039	168.039
259.696	259.696	274.973	274.973	290.249	290.249
412.459	412.459	412.459	412.459	427.735	443.011
244.42	244.42	259.696	259.696	259.696	274.973
458.288	473.564	473.564	473.564	473.564	473.564
183.315	183.315	183.315	198.591	198.591	213.868
259.696	259.696	259.696	274.973	274.973	274.973
213.868	213.868	213.868	229.144	229.144	229.144
213.868	213.868	213.868	213.868	213.868	229.144
274.973	274.973	290.249	290.249	290.249	305.525
273.0631	274.9726	278.7918	286.4299	290.2489	295.9776
106.934	122.21	137.486	152.763	168.039	183.315
91.657	106.934	106.934	122.21	122.21	137.486
30.553	45.829	45.829	45.829	61.105	61.105
45.829	45.829	61.105	61.105	61.105	76.381
122.210	122.210	137.486	152.763	152.763	152.763
122.21	122.21	137.486	152.763	152.763	168.039
121.368	137.338	136.628	151.178	152.598	166.438
91.657	106.934	122.21	137.486	137.486	152.763
45.829	45.829	45.829	61.105	61.105	78.381
76.381	91.657	91.657	91.657	106.934	106.934

85.4628	94.6980	102.2650	112.8859	117.6108	128.3605
488.84	519.393	519.393	549.945	549.945	565.221
259.696	259.696	274.973	274.973	274.973	274.973
458.288	458.288	458.288	458.288	473.564	473.564
397.183	397.183	412.459	412.459	412.459	427.735
274.973	290.249	290.249	305.525	305.525	305.525
290.249	290.249	305.525	305.525	305.525	305.525
213.868	213.868	213.868	213.868	213.868	229.144
183.315	183.315	183.315	183.315	198.591	229.144
290.249	305.525	305.525	320.801	320.801	320.801
412.459	427.735	412.459	427.735	443.011	458.288
326.912	334.5501	337.6054	345.2434	349.8262	358.992
458.288	473.564	488.84	504.116	504.116	519.393
351.354	366.63	381.906	381.906	381.906	397.183
351.354	366.63	381.906	381.906	397.183	397.183
336.078	351.354	366.63	351.354	366.630	381.806
351.354	366.63	366.63	381.906	381.906	381.906
274.973	290.249	305.525	320.801	336.078	351.354
244.420	244.420	244.420	259.696	259.696	274.973
229.144	229.144	229.144	244.420	244.420	259.696
290.249	305.525	336.078	336.078	336.078	336.078
320.801	320.801	336.078	351.354	351.354	351.354
320.8015	331.4947	343.7157	351.3537	355.9367	365.0926

51
397.34
244.42
488.84
290.249
366.63
488.84
445.947
336.078
381.906
427.735

386.7985

91.657
198.591
168.039
213.868
198.591
122.21
122.21
259.696
76.381
106.849

155.8092

183.315
290.249
458.288
274.973
488.84
213.868
290.249
229.144
229.144
305.525

303.6158

183.315
137.486
61.105
76.381
168.039
168.039
165.728
152.763
78.381
106.934

129.8171

565.221
274.973
473.564
427.735
305.525
320.801
229.144
229.144
336.78
473.564
363.6451

519.393
412.459
397.183
397.183
397.183
351.354
274.973
259.696
351.354
366.630
372.7408

屈光度（D）	组别	厚度	6	9	12	15
-3	Control	25/81	45.829	76.382	91.658	106.934
-5	Control	15/104	45.829	61.105	76.381	76.381
-5.625	Control	15/107	45.829	76.381	106.934	122.21
-5.75	Control	15/104	30.552	45.828	76.381	91.657
-6.375	Control	15/119	45.829	76.381	91.657	106.934
-6.625	Control	15/124	46.457	76.307	93.588	108.176
-8.25	Control	15/147	0	15.277	30.553	30.553
-5.8036			37.1893	61.0944	81.0217	91.8350
-4.5	5/2.5	15/85	15.276	15.276	30.553	45.829
-5.125	5/2.5	15/100	0	15.277	15.277	30.553
-5.625	5/2.5	15/106	15.277	30.553	45.829	76.381
-6.125	5/2.5	15/114	15.276	30.553	30.553	30.553
-6.75	5/2.5	15/127	45.828	76.381	106.933	122.21
-7.375	5/2.5	15/134	0	15.276	15.276	15.276
-5.9167			15.2762	30.5527	40.7368	53.4670
-3.5	5/5	25/93	45.828	91.657	122.21	168.039
-4.5	5/5	15/95	45.828	76.381	106.933	137.486
-4.875	5/5	15/98	76.381	137.486	168.038	183.315
-5.75	5/5	15/103	45.828	76.381	106.934	122.21
-6.25	5/5	15/109	15.276	45.828	61.104	76.381
-6.875	5/5	15/133	30.553	61.105	91.658	122.21
-7.625	5/5	15/144	30.552	61.104	76.381	106.933
-5.6250			41.4637	78.5631	104.7511	130.9391
-4.25	10/5	15/88	15.276	15.276	30.553	45.829
-4.875	10/5	15/103	0	0	15.277	15.277
-5.125	10/5	15/110	0	0	15.277	15.277
-6.25	10/5	15/123	0	15.277	30.553	45.829
-6.5	10/5	15/120	14.55	13.84	29.1	44.359
-7	10/5	15/133	15.277	15.277	30.553	30.553
-8.125	10/5	15/148	15.276	15.276	15.276	15.276
-6.0179			8.6256	10.7066	23.7984	30.3429

18	21	24	27	30	33
106.934	122.21	137.487	137.487	137.487	152.763
106.934	122.21	137.487	152.763	152.763	152.763
152.763	168.039	168.039	183.315	198.591	198.591
91.657	106.933	122.21	122.21	137.486	137.486
122.21	137.486	152.762	152.762	152.762	168.039
124.111	122.765	138.699	138.699	153.287	153.961
45.829	61.105	61.105	76.381	91.658	106.934
107.2054	120.1069	131.1127	137.6596	146.2906	152.9339
61.105	76.381	91.657	106.934	122.21	137.486
45.829	61.105	61.105	76.381	91.658	91.658
76.381	91.658	106.934	122.21	137.487	152.763
45.829	45.829	61.105	61.105	76.381	76.381
152.762	183.315	183.315	198.591	213.867	213.867
15.276	30.553	45.829	45.829	45.829	45.829
66.1970	81.4735	91.6575	101.8417	114.5720	119.6640
183.315	213.867	229.144	244.42	259.696	274.972
168.038	168.038	183.315	198.591	213.867	229.143
198.591	213.867	229.143	229.143	244.42	259.696
137.486	152.762	168.039	183.315	183.315	198.591
91.657	106.933	122.21	137.486	137.486	152.762
137.487	168.039	183.315	183.315	198.592	198.592
122.21	137.486	152.762	152.762	168.038	183.315
148.3977	165.8560	181.1326	189.8617	200.7734	213.8673
45.829	61.105	76.381	91.657	91.657	106.934
30.553	45.829	45.829	61.105	61.105	76.381
15.277	15.277	15.277	30.553	30.553	30.553
61.105	61.105	76.381	91.658	91.658	106.934
43.649	42.94	59.619	74.169	89.429	105.398
30.553	45.829	61.105	61.105	76.381	76.381
15.2766	30.553	30.553	30.553	45.819	45.829
34.6061	43.2340	52.1636	62.9714	69.5146	78.3443

36	39	42	45	48	51
152.763	152.763	152.763	152.763	152.763	152.763
183.315	183.315	198.592	213.868	229.144	229.144
215.868	213.868	213.868	229.144	229.144	229.144
137.486	137.486	137.486	137.486	152.762	152.762
168.039	183.315	183.315	198.591	198.591	198.591
153.287	183.137	199.745	215.006	215.68	231.614
106.934	122.21	137.487	137.487	152.763	152.763
159.6703	168.0134	174.7509	183.4779	190.1210	192.3973
137.486	152.763	168.039	183.315	198.591	198.591
122.21	122.21	137.487	137.487	152.763	152.763
152.763	168.039	183.315	198.592	198.592	198.592
91.657	91.657	106.934	106.934	122.21	122.21
229.143	229.143	229.143	229.143	229.143	229.143
61.105	61.105	61.105	61.105	76.381	76.381
132.3940	137.4862	147.6705	152.7627	162.9467	162.9467
274.972	290.249	305.525	320.801	320.801	336.077
229.143	244.42	244.42	259.696	259.696	259.696
259.696	259.696	259.696	274.972	290.248	305.525
198.591	213.867	213.867	213.867	229.144	229.144
152.762	152.762	168.038	168.038	183.315	183.315
198.592	198.592	213.868	213.868	213.868	213.868
183.315	183.315	183.315	183.315	198.591	198.591
213.8673	220.4144	226.9613	233.5081	242.2376	246.6023
122.21	137.486	152.763	168.039	183.315	183.315
91.658	91.658	106.934	106.934	122.21	122.21
30.553	45.829	45.829	45.829	61.105	61.105
106.934	122.21	137.487	137.487	152.763	152.763
121.368	120.658	135.208	136.628	150.468	149.758
91.658	106.934	122.21	122.21	137.487	137.487
61.105	61.105	76.381	76.381	97.657	91.657
89.3551	97.9829	110.9731	113.3583	129.2864	128.3279
